# Alignment of Australian Hospital Nutrition Standards With National and International Evidence‐Based Guidelines: A Scoping Review

**DOI:** 10.1111/jhn.70301

**Published:** 2026-06-29

**Authors:** Elizabeth Edwards, Tsun Lok Morris Tsang, Priya Iyer

**Affiliations:** ^1^ Nutrition and Dietetics, Susan Wakil School of Nursing and Midwifery, Faculty of Medicine and Health University of Sydney Sydney New South Wales Australia; ^2^ Charles Perkins Centre University of Sydney Sydney New South Wales Australia

**Keywords:** Australian dietary guidelines, ESPEN, foodservices, hospital meals, hospital nutrition, inpatient care, malnutrition, nutrition standards

## Abstract

Hospital malnutrition remains highly prevalent in Australia and contributes to poorer clinical outcomes and increased healthcare costs. Hospital nutrition standards play a critical role in ensuring nutritionally adequate menus and supporting patient intake. While Australian jurisdictions have developed hospital nutrition standards, the extent of their consistency and alignment with national and international evidence‐based guidelines has not been systematically examined. This scoping review aimed to identify and compare existing hospital nutrition standards across Australia and assess their alignment with the Australian Dietary Guidelines (ADG) and European Society for Clinical Nutrition and Metabolism (ESPEN) hospital nutrition guidelines. Guided by JBI methodology for scoping reviews and the Preferred Reporting Items for Systematic Reviews and Meta‐Analyses Extension for Scoping Reviews (PRISMA‐ScR) reporting guidelines, grey literature searches were conducted across government websites, national repositories, and targeted Google searches. Twelve documents met inclusion criteria, including six nutrition standards and six supporting documents. Directed content analysis guided by ADG and ESPEN deductive frameworks was used to extract and compare data across jurisdictions. Findings showed strong alignment with the ADG, particularly in five food group provision and macronutrient targets. Alignment with ESPEN guidelines was more variable. All jurisdictions met minimum energy and protein targets and offered patient menu choices; however, inconsistencies were observed in therapeutic diet provisions, macronutrient distribution, food service considerations, adaptations for diverse patient groups and monitoring practices. Hospital nutrition standards in Australia are fragmented. Developing a unified evidence‐based standard integrating ADG principles with ESPEN hospital nutrition recommendations could enhance consistency, quality, and equity in hospital nutrition care.

## Summary

- Australian hospital nutrition standards generally aligned well with the Australian dietary guidelines, particularly for five food group provision and minimum nutrient targets.
- Alignment with ESPEN hospital nutrition guidelines was inconsistent, with notable variation in therapeutic diets, macronutrient distribution and monitoring practices across jurisdictions.
- Although all jurisdictions met minimum energy and protein requirements and provided menu choice, the absence of a unified standard contributed to fragmented and inconsistent nutrition care practices across Australia.

## 1 Introduction

Adequate nutrition is fundamental for quality hospital care and is integral to patient recovery, contributing to reduced complications and improving clinical outcomes [1, 2]. Malnutrition is highly prevalent in hospitals with up to 40% of patients either malnourished or at risk of developing malnutrition during their admission [1, 2]. Malnutrition is associated with increased healthcare costs driven by longer length of stays, and higher hospital readmission rates and poorer overall health outcomes [1, 2]. Therefore, the provision of effective and evidence‐based nutrition care in hospitals is critical.

In response to the hospital malnutrition, Australian jurisdictions have developed hospital nutrition standards intended to ensure that menus meet minimum nutritional targets and cater to diverse therapeutic and cultural needs of patients [3]. The Australian Commission on Safety and Quality in Health Care (ACSQHC) has also incorporated nutrition into its national hospital accreditation standards reflecting its importance in patient safety and care quality. These initiatives were introduced to minimise inconsistencies in health care delivery and reduce variation in patient care across Australia [4]. However, there currently exists no unified national hospital food and nutrition standard or policy in Australia. Instead, hospitals operate under guidelines set by their state or territory, leading to variability in practices.

The Australian Dietary Guidelines (ADG) [5] provide a national evidence‐based framework promoting health and wellbeing in the general population but do not specifically address the nutritional needs of hospitalised populations. Conversely, the European Society for Clinical Nutrition and Metabolism (ESPEN) hospital nutrition guidelines offer internationally recognised evidence‐based recommendations for clinical nutrition practice tailored to hospital settings, including guidance on menu planning, nutrition targets and patient‐centred nutrition care [6]. The extent to which the various Australian hospital nutrition standards align with these frameworks has not been systematically examined. Given the importance of consistency, quality, and evidence‐based practice in healthcare delivery, mapping and evaluating existing hospital nutrition standards across Australia is both timely and necessary.

Therefore, this scoping review aimed to map and compare hospital nutrition standards across Australian public hospitals and assess their alignment with the ADG [5] and ESPEN hospital nutrition standards [6].

## 2 Methods

This review was guided by the JBI methodology for scoping reviews [7, 8] with additional adaptations for grey literature reviews in nutrition [9, 10]. Reporting followed the Preferred Reporting Items for Systematic Reviews and Meta‐Analyses Extension for Scoping Reviews (PRISMA‐ScR) [11]. Preliminary searches in MEDLINE yielded no relevant results as the review involves government documents available in relevant grey literature repositories only. Hence, the review was limited to grey literature sources only. As the review utilised only publicly available documents and did not involve any human participants, ethics approval was not required. The protocol was registered with the Open Science Framework (https://osf.io/6xa3r/).

### 2.1 Search and Sources

The search strategy targeted published grey literature, focussing on documents developed, published or endorsed by Australian federal, state and territory governments, health departments, clinical networks, and/or national nutrition and health quality organisations. A predefined list of all Australian federal, state and territory health departments and their associated nutrition/relevant pages was compiled to enable searching. Each website was screened using the same structured approach, including review of the first 20 search results per site ensuring consistency. Search sources included:

- Google (first 10 pages) and Google advanced search (site of domain: gov.au) (first 10 pages of results)
- Government health department websites (first 20 results per site)
- National repositories—Trove and Australian Commission on Safety and Quality in Health Care (ACSQHC) (first 10 pages)
- Snowballing of reference lists within documents determined eligible for full text review

Screening the first 10 pages of Google and repository results, and the first 20 results per government website provided a feasible yet comprehensive approach to identifying relevant documents. To reduce missing eligible material, these searches were complemented by multiple targeted strategies outlined ensuring broad coverage of existing Australian hospital nutrition standards. Handsearching of these repositories was conducted systematically.

Search terms were adapted for each source using Boolean operators (OR, AND) as below.

- *Google and Google advanced searches: (Hospitals OR inpatients OR institutions OR healthcare OR “health facility”) AND (Nutrition OR menu OR food OR meals OR dining) AND (Standards OR Guidelines OR framework OR recommendations)*
- *Government health department websites: “nutrition standards”*
- *National repositories: “hospital nutrition standards”*

Documents were eligible if they were published in English, available publicly online, applicable to inpatient care in Australian hospitals. No limits were placed on publication date, and patient age/demographics. To ensure that only current standards were included, all documents were assessed for indicators of currency, including publication and revision dates, version numbers and statements regarding updates or replacement. Where multiple versions of a document existed, the most current version was included in the review. Crosschecking across government health department websites and repositories was used to verify whether earlier versions had been superseded. Initial searches were carried out between 19 August 2025 to 3 September 2025.

### 2.2 Screening and Study Selection

Two reviewers (EE and MT) independently screened titles and summaries guided by the eligibility criteria (Supporting Information S1: Supplement 1), followed by full‐text review of potentially eligible documents. For documents without structured abstracts, document title and the most informative first page contents were considered as titles and summaries. The executive summary was examined when provided, or alternatively the introductory section or first one to two pages, to determine relevance at the initial screening stage. Discrepancies were resolved through discussion with unresolved disagreements resolved by a third reviewer (PI). A PRISMA‐ScR flow diagram [12] was used to document the study selection process.

## 3 Data Extraction

A data extraction template, structured as categorisation matrix was developed based on directed content analysis framework as outlined below. Extracted data included: jurisdiction (state/territory), document type, year, source; hospital focus (such as acute, mental health); nutrient targets (reference person, macronutrients, micronutrients); five food groups (vegetables, fruit, grains (cereals), meat and alternatives, dairy and alternatives, fats and oils/spreads, fluids); patient menu choices (meal planning guidance, meal occasion choice and variety, variation based on length of stay, cultural adaptations, catering for specific demographics, special diets); foodservice considerations (staffing, sustainability, menu management systems, mealtime environment); monitoring and evaluation (standards, menu/diets, patient nutrition care, clinical nutrition care); alignment with ADG [5] and ESPEN hospital nutrition guidelines [6].

## 4 Data Analysis

Directed content analysis [13] was employed as it is well‐suited to the systematic examination of policy standards and guidelines when existing theoretical frameworks can guide the coding structure. The unit of analysis comprised key components of the hospital nutrition standards and associated documents for each Australian jurisdiction.

### 4.1 Preparation Phase

Deductive coding categories were devised by familiarising with the data and guided by relevant recommendations from the ADG [5], Nutrient Reference Values for Australia and New Zealand [14], and ESPEN hospital nutrition guidelines [6]. These were mapped to the main domains and subdomains (Supporting Information S1: Supplements 2 and 3).

### 4.2 Organisation Phase

A structured categorisation matrix with pre‐defined themes/domains was and piloted by two reviewers (E.E. and M.T.). Data from nutrition standards and associated documents were extracted into the matrix. Additional inductive codes were added as they emerged during analysis (Supporting Information S1: Supplement 4).

### 4.3 Reporting Phase

A comparison matrix table summarised degree of alignment (fully aligned, partially aligned, not aligned or unable to determine alignment) with ADG [5] and ESPEN hospital nutrition guidelines [6] across jurisdictions based on pre‐defined domains, identifying gaps and inconsistencies. A coding framework guided this step ensuring consistency in ascertaining alignment (Supporting Information S1: Supplement 5).

## 5 Comparative Content Analysis

The comparison matrix was used to examine similarities and differences across jurisdictional hospital nutrition standards and associated documents. Alignment with the ADG [5] and ESPEN hospital nutrition guidelines [6] were categorised as: fully aligned; partially aligned; not aligned; unable to determine. A jurisdiction was defined as having a strong alignment to a domain when all components were aligned with only a maximum of one subcategory being partially aligned. A jurisdiction was defined as having moderate alignment to a domain when a maximum of two subcategories were partially aligned, and a maximum of one subcategory was not aligned. A jurisdiction was defined as weakly aligned to a domain when three or more subcategories were partially aligned, and two or more subcategories were not aligned.

## 6 Results

**Figure 1:**
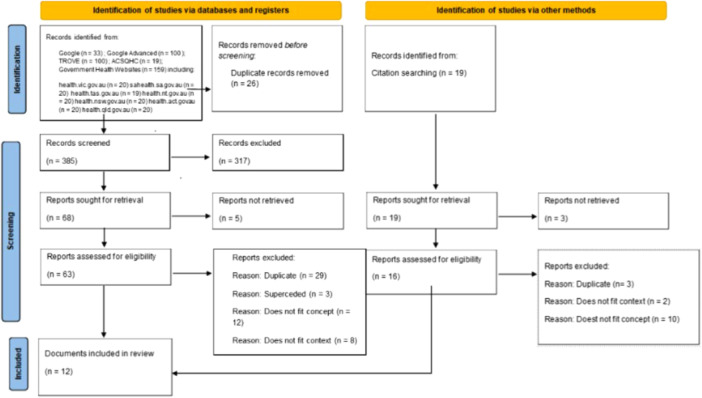
Preferred Reporting Items for Systematic Reviews and Meta‐Analyses Extension for Scoping Reviews (PRISMA‐ScR) flow diagram. *Source:* Page MJ, et al. BMJ 2021;372:n71. doi: 10.1136/bmj.n71. This work is licensed under CC BY 4.0. To view a copy of this license, visit https://creativecommons.org/licenses/by/4.0/ .

A total of 430 records were identified through grey literature searches, including 19 documents identified through snowballing reference lists. After removal of duplicates, 404 records were screened for eligibility. Following full‐text review, 12 records were eligible for inclusion in the review and analysis (Figure 1; Supporting Information S1: Supplements 6 and 7). Of the 12 documents included in this review, six were nutrition standards and six were associated supporting documents. All were endorsed by state and territory governments and outlined specific requirements for menu planning and nutrition care in public hospitals. Most publications were from New South Wales (NSW) (*n* = 3) [15, 16, 17] and South Australia (SA) (*n* = 3) [18, 19, 20], followed by Queensland (QLD) (*n* = 2) [21, 22], Western Australia (WA) (*n* = 2) [23, 24], Victoria (VIC) (*n* = 1) [25, 26], and Australian Capital Territory (ACT) (*n* = 1) [27]. As the ACT standards [27] explicitly refer to following the NSW nutrition standards [15, 16, 17], they were excluded from the comparative content analysis in subsequent phases of the review. No eligible documents were identified for Tasmania or the Northern Territory (Supporting Information S1: Supplement 8).

## 7 Alignment With the ADG

All included hospital nutrition standards [15, 16, 17, 18, 19, 20, 21, 22, 23, 24, 25, 26] aligned with the ADG [5] daily serves of five food groups (vegetables and legumes, fruits, grain (cereals) foods, lean meats and poultry, fish, eggs, tofu, nuts and seeds, dairy and alternatives) by specifying the recommended daily serves based on reference person's age and sex (Table 1; Supporting Information S1: Supplement 9). SA and WA standards [18, 24] applied nutrient targets to adult populations only while SA referenced NSW standards for paediatric targets. Four jurisdictions (NSW, VIC, SA, WA) [15, 16, 17, 18, 19, 20, 23, 24, 25, 26] fully aligned with the Nutrient Reference Values' macronutrient recommendations [14] for saturated fat, dietary fibre and fluid intake with quantitative targets for minimum requirements. QLD standards [21] partially aligned, as fluid requirements were not specified quantitatively. Four jurisdictions [15, 16, 17, 18, 19, 20, 23, 24, 25, 26] fully aligned with the Nutrient Reference Values' micronutrient recommendations [14] specifying targets for vitamin C, folate, calcium, iron, zinc and sodium. QLD [21] did not specify micronutrient targets and was therefore not aligned. Supporting Information S1: Supplement 5 outlines the criteria for determining each jurisdictions degree of alignment (fully, partially, not aligned or unable to determine alignment for each category.

**Table 1:** Comparative analysis of nutrition standards and associated documents' alignment with Australian Dietary Guidelines, by jurisdiction.

	New South Wales	Queensland	Victoria	South Australia	Western Australia
Five Food groups	✓	✓	✓	✓	✓
*Nutrient Adequacy—macronutrients*					
Saturated Fats, Dietary Fibre, Fluids	✓	**P**	✓	✓	✓
*Nutrient Adequacy—micronutrients*					
Vitamin C, Folate, Calcium, Iron, Zinc, Sodium	✓	✗	✓	✓	✓

Abbreviations: ✓ = fully aligned, **P** = partially aligned, ✗ = not aligned, **−** = unable to be determine alignment.

## 8 Alignment With ESPEN Hospital Nutrition Guidelines

### 8.1 Nutrient Adequacy

Alignment with ESPEN recommendations [6] varied considerably across domains and jurisdictions (Table 2; Supporting Information S1: Supplement 9). All five jurisdictions [15, 16, 17, 18, 19, 20, 21, 22, 23, 24, 25, 26] fully aligned with ESPEN recommendations [6] for minimum energy (25 kcal/kg actual body weight/day) and protein (0.8–1 g/kg actual body weight/day) requirements for the standard hospital diet. Although QLD's short‐stay menu [21] (for patients not at nutritional risk and admitted for < 7 days) energy targets fell below ESPEN recommendations [6], the long‐stay menu was considered the standard diet for the purposes of this review. Across jurisdictions, while energy and protein targets were broadly comparable, the reference persons used to derive these targets were varied. None of the jurisdictions fully aligned with ESPEN recommendations [6] for macronutrient distribution for standard hospital menus (carbohydrate 50%–60%, lipids 35%–40%, protein 15%–20%); VIC [25] and SA [18] specified macronutrient ranges aligned with ADG acceptable macronutrient distribution range [5] rather than ESPEN recommendations [6]. In contrast, others (NSW, Qld and WA) [15, 16, 17, 21, 22, 23, 24] did not specify any macronutrient distribution ranges (Table 3).

**Table 2:** Comparative analysis by jurisdiction of nutrition standards and associated documents alignment with ESPEN hospital nutrition guidelines.

	New South Wales	Queensland	Victoria	South Australia	Western Australia
*Nutrient adequacy—macronutrients*					
Protein and Energy	✓	✓ a	✓	✓	✓
Proportion of carbohydrates, lipids and proteins	—	**—**	✗	✗	**—**
*Patient choices*					
Minimum two main meal choices (lunch and dinner)	✓	✓ a	✓	✓	✓
Systematic mid‐meal service (snacks)	✓	✓	✓	✓	✓
Minimum diets offered	✓	**P**	**P**	✓	✓
Therapeutic Diets references	✓	**P**	**P**	✓	✓
Texture Modified Diets and dysphagia screening mentioned	**P**	**P**	✓	✓	✓
Vegetarian options (protein and energy)	✓	✓	✓	✓	✓
No separate vegan diet	✗	✓	✓	✗	✓
Accommodating religious/cultural/food preferences	✓	✓	✓	✓	**P**
*Food service considerations*					
Staffing roles and responsibilities	**—**	**P**	✓	**P**	✓
Food Service Dietitian	✗	✓	✓	✓	✓
Sustainability	✓	✓	✓	✗	✗
Adapted to different patient groups including long stay rehab and palliative	✓	**P**	**P**	✓	**P**
Protected mealtimes	✓	✓	✗	✓	✓
Menu Management System	✓	✓	**—**	✓	**—**
*Monitoring and evaluation*					
Full Menu review 3–5 yrs	✓	✓	✓	✓	✓
Patient satisfaction surveys annually	**P**	✓	✓	**P**	✓
Patient intake monitoring mentioned	**P**	✗	✗	**P**	**P**
References nutrition screening and assessment associated document	✓	**P**	✗	✓	✓

Abbreviations: ✓ = fully aligned, **P** = partially aligned, ✗ = not aligned, **−** = unable to be determine alignment.

^a^ QLD short stay menu (for patients not at nutritional risk admitted for less than 7 days) energy targets and minimum choice and main meals were below ESPEN recommendations).

**Table 3:** Comparison of adult nutrition standards' reference person and minimum energy and protein targets in the hospital standard diet across jurisdictions.

	Victoria	South Australia	Western Australia	Queensland	New South Wales
Reference person	Male 51–70 years old Weight 80 kg Height 174 cm BMI 26.4 kg/m2	Male 70+ years old Weight 76 kg PAL 1.2 Female 19–50 years old Weight 65 kg	Male 51–70 years old Weight 76 kg	Male 65 years old Weight 76 kg	Weight 76 kg^a^
Minimum energy	8500 kJ	8000 kJ	8000 kJ	8000 kJ	8000 kJ
Minimum protein	85 g	90 g	90 g	91 g	90 g

*Note:* Standard diet in hospitals refers to the diet provided to most inpatients on admission who are at low or no nutritional risk and do not require special diets.

a Age group not mentioned. For consistency, male and female of 19–50 years old were used as reference when determining minimum requirements for domains including core food group serves and micronutrients.

### 8.2 Patient Menu Choices and Diet Variety

All five jurisdictions [15, 16, 17, 18, 19, 20, 21, 22, 23, 24, 25, 26] fully aligned with ESPEN recommendations [6] by offering a minimum of two menu choices at main meals (lunch and dinner) and providing structured mid‐meal services. Standards also commonly addressed vegetarian options, therapeutic diet options (such as high protein diets) and texture‐modified diets including adaptations for specific patient groups (e.g., mental health, paediatrics, maternity, allergy/intolerances, cultural). However, only NSW, SA and WA [16, 18, 24] referenced therapeutic diet specifications [17] to design adapted therapeutic and texture‐modified menus. NSW, QLD, VIC, SA [15, 16, 17, 18, 19, 20, 21, 22, 25, 26] also fully aligned with ESPEN recommendations [6] by providing guidance on accommodating religious beliefs and food preferences while WA [23, 24] only referred to these as an overarching principle of the standards and was therefore partially aligned. Jurisdiction‐specific deviations were limited. In QLD [21, 22], the short‑stay menu did not meet the minimum choice requirements, although the long‑stay menu did. Other differences between jurisdictions related primarily to the level of detail provided rather than substantive divergence from core standards. For example, QLD and VIC [21, 22, 25, 26] were partially aligned as they either did not meet or specify the nutrient targets when offering a hospital diet for nutritionally at risk/malnourished patients (30 kcal energy/kg actual body weight/day and 1.2 g protein/kg actual body weight/day) [15, 16, 17, 18, 19, 20, 21, 22, 23, 24, 25, 26] [16, 17, 18, 24]. Although all jurisdictions offered texture‐modified diets, NSW and QLD [16, 21, 22] did not reference dysphagia screening by a speech pathologist resulting in partial alignment with ESPEN recommendations [6]. Interestingly, only VIC [25] standards referred to 14‐h rule which specify recommended limit of overnight fasting (Supporting Information S1: Supplement 9).

### 8.3 Foodservice Considerations

Overall, all five jurisdictions [15, 16, 17, 18, 19, 20, 21, 22, 23, 24, 25, 26] demonstrated partial alignment with ESPEN foodservice recommendations [6]. VIC and WA [23, 24, 25, 26] clearly outlined responsibilities for food production and delivery while QLD, VIC, SA and WA [18, 19, 20, 21, 22, 23, 24, 25, 26] specified the requirement for dietitian oversight of menu planning and implementation. However, NSW standards did not define foodservice roles rendering alignment undeterminable. NSW, QLD and VIC [15, 16, 17, 21, 22, 25, 26] emphasised the use of high‐quality, sustainable food ingredients minimising food waste in line with ESPEN recommendations [6]. NSW and SA [15, 16, 17, 18, 19, 20] addressed adaptations to food delivery for acute, rehabilitation and palliative care while other jurisdictions (QLD, VIC, WA) [21, 22, 23, 24, 25, 26] referenced at least one [6]. All jurisdictions except VIC [15, 16, 17, 18, 19, 20, 21, 22, 23, 24] stated mealtimes should be protected, supporting ESPEN recommendations' [6] emphasis on the mealtime environment. NSW, QLD and SA [15, 16, 17, 18, 19, 20, 21, 22] were fully aligned with ESPEN recommendations [6] for structured food ordering systems.

## 9 Monitoring and Evaluation

All jurisdictions [15, 16, 17, 18, 19, 20, 21, 22, 23, 24, 25, 26] aligned with ESPEN recommendations [6] to review menus every 3‐5 years. While all referenced patient satisfaction surveys, only QLD, VIC and WA [21, 22, 23, 24, 25, 26] specified a minimum annual review frequency in line with ESPEN recommendations [6]. NSW, SA and WA [15, 16, 17, 18, 19, 20, 23, 24] partially aligned by referencing nutrition screening and monitoring documents without detailing protocols. While specific details on clinical nutrition care were beyond the scope of this review, the presence of references to supporting documents was considered indicative of system‐level support.

## 10 Comparative Analysis Across Jurisdictions

Comparative synthesis highlighted consistent strengths in alignment with the ADG [5] and Nutrient Reference Values [14], and variable alignment with ESPEN hospital nutrition guidelines [6] (Tables 1 and 2). For criteria defining a jurisdiction strength of alignment to a domain (strong, moderate or weak) refer to the comparative content analysis in methods section. Strong alignment was observed across all five jurisdictions [15, 16, 17, 18, 19, 20, 21, 22, 23, 24, 25, 26] for ADG five food groups' recommendations [5] and ESPEN minimum protein and energy recommendations [6]. Moderate alignment was observed for ADG nutrient adequacy [5] in QLD [21, 22], and for ESPEN patient choice [6] in NSW, VIC and SA [15, 16, 17, 18, 19, 20, 25, 26]. All jurisdictions [15, 16, 17, 18, 19, 20, 21, 22, 23, 24, 25, 26] demonstrated moderate alignment with ESPEN [6] food services recommendations. Weak alignment was identified across all jurisdictions [15, 16, 17, 18, 19, 20, 21, 22, 23, 24, 25, 26] for ESPEN macronutrient recommendations [6]. QLD [21, 22] demonstrated weak alignment with ESPEN [6] patient choice and VIC [25, 26] demonstrated weak alignment with monitoring and evaluation domain.

## 11 Discussion

This is the first scoping review mapping and comparing hospital nutrition standards across Australian public hospitals and evaluating their alignment with the ADG [5] and ESPEN hospital nutrition guidelines [6]. Evaluating hospital nutrition standards across Australian public hospitals is critical to ensuring equity in nutrition care nationally and supporting efforts to reduce hospital malnutrition and its associated adverse clinical and economic outcomes [1, 2, 3]. Overall, the comparative analysis demonstrated strong and consistent alignment with the ADG [5] across jurisdictions, particularly for five food group provision and nutrient adequacy. In contrast, alignment with ESPEN hospital nutrition guidelines [6] was variable highlighting important gaps in the application of international evidence‐based standards specific to hospitalised patients.

The strong alignment observed between Australian hospital nutrition standards [15, 16, 17, 18, 19, 20, 21, 22, 23, 24, 25, 26] and the ADG [5] reflects the widespread adoption of national dietary guidance across jurisdictions. This consistency is likely influenced by the ADG's [5] role as Australia's primary nutrition framework and its integration into public health and clinical settings. All jurisdictions except QLD [15, 16, 17, 18, 19, 20, 23, 24, 25, 26] also explicitly specified meeting recommended daily intake (RDI) [14] for key micronutrients (vitamin C, folate, calcium, iron and zinc). While QLD [21, 22] did not specify quantitative micronutrient targets, menu analysis was used to assess whether RDIs [14] were met for the reference person [21]. This suggests that, in principle, standard hospital diets across jurisdictions are designed to meet nutrient requirements for patients whose needs do not exceed those of the reference person. However, inconsistencies were identified in how jurisdictions addressed situations where the reference person's micronutrient requirements for example were lower than those of another sex or age group which raise concerns that some patient groups may not consistently meet nutrient adequacy (Supporting Information S1: Supplements 2 and 9). Further, it must be noted that ADG [5] is designed for the general population and does not account for the increased metabolic demands, disease related needs and functional limitations commonly experienced by hospitalised patients. Therefore, reliance on the ADG [5] alone may be inadequate to optimise nutrition care in hospital settings.

ESPEN hospital nutrition guidelines [6] were applied alongside ADG [5] to determine whether hospital‐specific nutrition requirements were met. While ESPEN [6] do not specify minimum micronutrient targets for general inpatient populations, it provides minimum daily protein and energy targets reflecting the increased metabolic demand of hospital patients. All jurisdictions' [15, 16, 17, 18, 19, 20, 21, 22, 23, 24, 25, 26] standard diets met ESPEN's [6] minimum energy targets and exceeded minimum daily protein targets. Nevertheless, variation in reference body weight assumptions resulted in differences in nutrient targets between jurisdictions highlighting how inconsistencies in underlying assumptions can translate into variation in patient care, even when standards are nominally aligned. Variation in macronutrient alignment warrants particular attention. Although no jurisdiction fully aligned with ESPEN macronutrient distribution recommendations [6], the reasons differed. VIC [25, 26] and SA [18, 19, 20] adopted ADG macronutrient ranges [5], reflecting an emphasis on local national dietary guidance. In contrast, other jurisdictions [15, 16, 17, 21, 22, 23, 24] did not specify macronutrient distributions, limiting the ability to assess alignment. This distinction is important, as non‐alignment due to substitution of an alternative evidence‐based framework differs conceptually from non‐alignment due to omission. Future national harmonisation efforts may benefit from clearer articulation of the rationale for macronutrient targets and greater consistency in their specifications.

Cross‐jurisdictional comparison was further complicated by inconsistent terminology and definitions of “standard” and “short‐stay” menus [15, 16, 17, 18, 19, 20, 21, 22, 23, 24, 25, 26]. While jurisdictions defined short‐stay admissions as 3 days or less [16, 24, 25], QLD defined short‐stay as < 7 days [21, 22], consistent with ESPEN hospital nutrition guidelines [6] for a standard diet. Although QLD's long stay menu met ESPEN [6] minimum nutrient targets for the standard diet, energy targets for nutritionally at‐risk patients were unmet, while the short‐stay menu fell below these energy recommendations [21, 22]. A key consideration when interpreting differences in nutrient targets across jurisdictions is the variation in the reference persons used to derive energy and nutrient requirements. Because recommended intakes scale with age, sex and body weight, jurisdictions that base targets on different reference assumptions will naturally produce different absolute values, even when their underlying nutritional intent is similar. As a result, some apparent differences in nutrient targets may reflect methodological variation rather than substantive divergence in standards. Such variability in reference persons and derivation methods suggests inconsistencies in how these targets are operationalised which may have implications for comparability and implementation. Meeting nutrient targets alone does not ensure adequate intake, as hospital patients frequently consume less than the prescribed meals [1, 28, 29, 30]. Well‐documented barriers to intake including poor appetite, acute illness, fatigue, dysphagia, pain, constipation, nausea, restrictive diet orders, food preferences, inadequate feeding assistance and mealtime interruptions [1, 28, 29, 30] highlight the importance of incorporating supportive strategies to enhance intake within the standards [1, 6, 28, 29, 30]. Clinically meaningful practices identified in individual jurisdictions warrant broader consideration in future national standard‐setting. For example, VIC's [25, 26] 14‐h rule, which limits the overnight fasting interval between the evening meal and breakfast may reflect a clinical principle aimed at reducing unintentional fasting and supporting adequate energy intake although fasting times in clinical settings vary widely [31]. Although not universally adopted, this type of operational specification illustrates how some jurisdictions provide more detailed guidance on practices that directly influence nutritional adequacy and patient experience underscoring opportunities for shared learning and potential harmonisation across Australia's hospital nutrition standards.

Although all jurisdictions [15, 16, 17, 18, 19, 20, 21, 22, 23, 24, 25, 26] met ESPEN recommendations [6] for meal choice and mid‐meal provision, substantial variation existed in how texture‐modified, and therapeutic diets were addressed. Only NSW, SA and WA [15, 16, 17, 18, 19, 20, 23, 24] included or referenced established guidelines on the development of therapeutic diets (Table 2), indicating the strong alignment with the ESPEN hospital nutrition guidelines [6]. However, QLD and VIC [21, 22, 25, 26] mentioned therapeutic diets but did not reference any guidelines informing these diets (Table 2). The lack of structured guidance in therapeutic and texture‐modified diets could lead to reduced quality and inconsistencies in nutrition care delivery as these diets require precise nutrient or texture specifications along with the coordinated input from multidisciplinary teams [6] regarding hospital food service management across jurisdictions, particularly in staffing requirements and resource allocation with only VIC and WA [23, 24, 25, 26] detailing staff roles and responsibilities which could lead to variability in service delivery across jurisdictions. Hospitals with limited resources or workforce capacity, such as remote and rural hospitals [31] would be affected disproportionally leading to disparities in nutrition care delivery. Absence of such details could also affect the sustainability and monitoring of the foodservice systems especially given that the responsibilities for reviewing and reporting on these metrics are not defined uniformly within the standards menus [15, 16, 17, 18, 19, 20, 21, 22, 23, 24, 25, 26].

Similar inconsistency was observed in other foodservice considerations. While all jurisdictions [15, 16, 17, 18, 19, 20, 21, 22, 23, 24, 25, 26] required periodic menu review, fewer specified regular patient satisfaction surveys or monitoring of intake and nutrition status. These processes are critical for early identification of nutritional risk and prevention of hospital malnutrition [2, 6, 28, 30, 32]. The lack of consistent detail in this domain may reflect the complexity of food service governance and the division of responsibilities between health departments, hospitals, and external providers. Religious, cultural, and food preference considerations were generally well addressed, reflecting increasing recognition of cultural safety and person‐centred care within Australian healthcare systems. However, variability in the depth and operationalisation of these considerations suggests opportunities for further standardisation. A notable challenge encountered during this review was the limited public accessibility of several key operational documents, including diet manuals, foodservice management systems and detailed menu specifications. In multiple jurisdictions, these materials were referenced within standards but not publicly available, preventing assessment of their content or alignment with national or international guidance. This lack of transparency restricts the ability to evaluate system performance, compare practices across jurisdictions or identify opportunities for harmonisation. Greater public availability of supporting documents would enhance accountability, facilitate cross‐jurisdictional learning and strengthen the evidence base underpinning hospital nutrition standards.

As no published studies to date have evaluated the success and impact associated with the implementation of these jurisdictional nutrition standards, it restricts the understanding of whether current standards are achieving their intended goals, such as improving the nutrition intake of patients and reducing the burden of malnutrition. Limited reference to clinical nutrition care pathways and intake monitoring may also reflect the intended scope of menu standards; however, these omissions risk undermining the effectiveness of nutrition care systems if food provision is not linked to patient‐level outcomes. Stronger integration between menu standards, nutrition screening, and monitoring frameworks would support more comprehensive nutrition governance and align with international best practice recommendations [6].

The variability identified across jurisdictions reflects Australia's fragmented healthcare governance structure, in which responsibility is shared between federal, state, and territory governments [4, 33]. This fragmentation may have contributed to duplication in standard development and variable interpretation of best‐practice guidance [3, 30, 33]. Developing a national hospital food and nutrition standard, informed by both the ADG [5] and ESPEN hospital nutrition guidelines [6], could reduce unwarranted variation, improve consistency, and strengthen nutrition governance across Australian public hospitals. Such a standard could provide clear guidance on nutrient targets, diet definitions, patient choice, and monitoring processes while allowing flexibility for local implementation. Such an approach aligns with broader national and international trends toward standardisation in healthcare quality and with Dietitians Australia's national competency standards, which recognise dietitians as central to hospital malnutrition prevention [1, 34].

## 12 Strengths and Limitations

This review is strengthened by its systematic methodology, comprehensive grey literature search, and adherence to JBI and PRISMA‐ScR guidelines (Supporting Information S1: Supplement 10) [7, 8]. Independent screening and data extraction by two reviewers enhanced methodological rigour. The use of directed content analysis enabled structured comparison against established national and international frameworks. Limitations include reliance on publicly available documents, which may not capture local implementation practices or supplementary internal guidelines. In addition, the data analysis process relied highly on the comprehensiveness of each document. Thus, the absence of explicit information does not necessarily imply the absence of the practice, leading to an underestimation of the alignment with the ADGs [5] and the ESPEN hospital nutrition guidelines [6]. However, associated documents were snowballed from the reference lists to address this. Comparisons of nutrient targets across jurisdictions are limited by differences in the reference persons used to derive energy and nutrient requirements. Variation in assumed age, sex and body weight means that some differences in targets may reflect methodological choices rather than true differences in nutritional standards. Additionally, the review focused on documented standards rather than evaluating adherence or patient outcomes, and the assessment of alignment was necessarily interpretive despite the use of a predefined coding framework.

## 13 Conclusions

Australian hospital nutrition standards demonstrate strong alignment with national dietary guidance (ADG) but variable alignment with international, hospital‐specific nutrition recommendations (ESPEN). Although nutritional adequacy is consistently addressed across jurisdictions, important gaps persist in macronutrient distribution, hospital diet definitions, foodservice governance, and monitoring/evaluation processes. These findings highlight the limitations of relying on jurisdiction‐based standards alone and support the development of a unified, evidence‐based national hospital nutrition standard. Establishing such a standard would promote consistency, quality, and equity in nutrition care for hospitalised patients across Australia and strengthen national efforts to prevent hospital malnutrition.

## Author Contributions

Conceptualization: Priya Iyer. Methodology: Priya Iyer. Software: Priya Iyer. Validation: Priya Iyer, Elizabeth Edwards, and Tsun Lok Morris Tsang. Formal analysis: Elizabeth Edwards, and Tsun Lok Morris Tsang. Resources: Priya Iyer. Data curation: Elizabeth Edwards, Tsun Lok Morris Tsang, and Priya Iyer. Writing – original draft preparation: Elizabeth Edwards, and Tsun Lok Morris Tsang. Writing – review and editing: Priya Iyer. Visualization: Elizabeth Edwards, Tsun Lok Morris Tsang, and Priya Iyer. Supervision: Priya Iyer. Project Administration: Priya Iyer. All authors have read and approved the final manuscript for publication.

## Funding

The authors have nothing to report.

## Conflicts of Interest

The authors declare no conflicts of interest.

## Institutional Review Board Statement

The study was conducted according to the guidelines of the Declaration of Helsinki and no ethical approval was needed as it was a scoping review with no involvement of any human participants.

## Supporting information

Supporting File

## Data Availability

The data that support the findings of this study are available in the supplementary material of this article.
