# Supplementary material for: Alignment of Australian Hospital Nutrition Standards With National and International Evidence‐Based Guidelines: A Scoping Review
Source: J Hum Nutr Diet. 2026 Jun 29;39(4):e70301. doi: 10.1111/jhn.70301 (PMC13312438; doi:10.1111/jhn.70301)
Supplement: Supplementary file 1 — Supporting File [file JHN-39-0-s001.docx]

**Supplement 1: Inclusion Criteria**

| ***Criterion*** | **Description** |
| --- | --- |
| ***Participants*** | Not applicable as the focus is on policy, guidelines and standards documents, not individuals or patient populations |
| ***Concept*** | Hospital nutrition standards or menu planning guidelines and associated documents applicable to inpatients |
| ***Context*** | Australian public hospitals (acute, mental health, paediatric) |
| ***Sources*** | Grey literature only: government policies, clinical network documents, national frameworks/guidelines, official state health departments’ foodservice/menu guidelines |
| ***Exclusions*** | Peer-reviewed journal articles, internal/local protocols not publicly available, aged care setting or outpatient policies |

**Supplement 2: Alignment with the Australian Dietary Guidelines**^6^

| Number | Recommendation | Mapped: Main Category | Subcategory |
| --- | --- | --- | --- |
| 1 | Estimated energy requirements of adult using predicted BMR x PA. Schofield equation is used to calculate the BMR  Applied the same to children and adolescents | Nutrient Adequacy | Energy |
| 2 | **AI:**  0-6 months: 60g/day  7-12 months: 95g/day | Nutrient Adequacy | Carbohydrate |
| 3 | **AI**  **Infants:**  0-6months: 10 g (1.43 g/kg body weight)  7-12 months: 14 g (1.60 g/kg body weight)    **Children and Adolescents:**  1-3 yr  EAR: 12 g/day (0.92 g/kg)  RDI:14 g/day (1.08 g/kg)    4-8 yr  EAR: 16 g/day (0.73 g/kg)  RDI: 20 g/day (0.91 g/kg)    **Boys:**  9-13 yr  EAR: 31 g/day (0.78 g/kg)  RDI: 40 g/day (0.94 g/kg)    14-18 yr  EAR: 49 g/day (0.76 g/kg)  RDI: 65 g/day (0.99 g/kg)    **Girls:**  9-13 yr  EAR: 24 g/day (0.61 g/kg)  RDI: 35 g/day (0.87 g/kg)    14-18 yr  EAR: 35 g/day (0.62 g/kg)  RDI: 45 g/day (0.77 g/kg)    **Men:**  19-70 yr  EAR: 52 g/day (0.68 g/kg)  RDI: 64 g/day (0.84 g/kg)    >70 yr  EAR: 65 g/day (0.86 g/kg)  RDI: 81g/day (1.07 g/kg)    **Women:**  19-70 yr  EAR: 37 g/day (0.60 g/kg)  RDI: 46 g/day (0.75 g/kg)    >70 yr  EAR: 46 g/day (0.75 g/kg)  RDI: 57 g/day (0.94 g/kg)    **Pregnancy: (EAR & RDI)**  (2nd and 3rd trimesters)  14-18 yr: 47 g/day (0.82 g/kg) & 58 g/day (1.02 g/kg)  19-30 yr: 49 g/day (0.80 g/kg) & 60 g/day (1.00 g/kg)  31-50 yr: 49 g/day (0.80 g/kg) & 60 g/day (1.00 g/kg)    **Lactation: (EAR & RDI)**  14-18 yr: 51 g/day (0.90 g/kg) & 63 g/day (1.1 g/kg)  19-30 yr: 54 g/day (0.88 g/kg) & 67 g/day (1.1 g/kg)  31-50 yr: 54 g/day (0.88 g/kg) & 67 g/day (1.1 g/kg) | Nutrient Adequacy | Protein |
| 4 | **Infants:**  Age & AI  **0-6 months** - Total fat 31 g/day  n-6 polyunsaturated fats 4.4 g/day  n-3 polyunsaturated fats 0.5 g/day  **7-12 months** - Total fat 30 g/day  n-6 polyunsaturated fats 4.6 g/day  n-3 polyunsaturated fats 0.5 g/day | Nutrient Adequacy | Fat |
| 5 | Limit intake of Saturated fat, added salt, added sugar and alcohol;   10% of total energy intake | Nutrient Adequacy | Saturated Fat |
| 6 | **Linoleic acid; a-linolenic acid; Total LC n-3 (DHA+EPA+DPA)**  **Boys and girls**   1-3 yr: 5 g/day; 0.5 g/day; 40 mg/day  4-8 yr: 8 g/day; 0.8 g/day; 55 mg/day  **Boys**   9-13 yr: 10 g/day; 1.0 g/day; 70 mg/day  14-18 yr: 12 g/day; 1.2 g/day; 125 mg/day  **Girls**   9-13 yr: 8 g/day; 0.8 g/day; 70 mg/day  14-18 yr: 8 g/day; 0.8 g/day’ 85 mg/day  **Adults 19+ yr**   Men: 13 g/day; 1.3 g/day; 160 mg/day  Women: 8 g/day; 0.8 g/day; 90 mg/day | Nutrient Adequacy | Omega 6 & 3 |
| 7 | **Carbohydrate:** 45-65%  **Protein:** 15-25%  **Fat:** 20-35% | Nutrient Adequacy | AMDR |
| 8 | **Children and Adolescents: (AI)**  Boys/Girls:   1-3 yr: 14g/day  4-8 yr: 18g/day    Boys:  9-13 yr: 24g/day  14-18 yr: 28g/day    Girls:  9-13 yr: 20g/day  14-18 yr: 22g/day  Pregnancy: 25g/day  Lactation: 27g/day    **Adults: (AI)**  Men:  19+ yr: 30g/day    Women:  19+ yr: 25g/day  Pregnant: 28g/day  Lactation: 30g/day | Nutrient Adequacy | Dietary Fibre |
| 9 | **Water – AI**  **Infants:**  0-6 months: 0.7 L/day (from breast milk or formula)  7-12 months: 0.8 L/day (from breast milk, formula, food, plain water and other beverages, including 0.6 L as fluids)    **FLUID – AI**  **Children and adolescents:**  Boys/Girls:  1-3 yr: 1L/day  4-8 yr: 1.2L/day    Boys:  9-13 yr: 1.6L/day  14-18 yr: 1.9L/day    Girls:  9-13 yr: 1.4L/day  14-18 yr: 1.6L/day  Pregnancy: 1.8L/d  Lactation: 2.3L/d    **Adults:**  Men  19+ yr: 2.6L/day    Women:  19+ yr: 2.1L/day  Pregnancy: 2.3L/d  Lactation: 2.6L/d | Nutrient Adequacy | Fluid |
| 10 | **Infants: (AI)**  **0-6 months: 250 µg/day of retinol (as retinyl esters)**  **7-12 months: 430 µg/day of retinol equivalents (REs)**    **Children & Adolescents: (EAR & RDI)**  All   1-3 yr: 210 µg/day & 300 µg/day  4-8 yr: 275 µg/day & 400 µg/day  Boys   9-13 yr: 445 µg/day & 600 µg/day  14-18 yr: 630 µg/day & 900 µg/day  Girls   9-13 yr: 420 µg/day & 600 µg/day  14-18 yr: 485 µg/day & 700 µg/day    **Adults: (EAR & RDI)**  Men   19-30 yr: 625 µg/day & 900 µg/day  31-50 yr: 625 µg/day & 900 µg/day  51-70 yr: 625 µg/day & 900 µg/day  >70 yr: 625 µg/day & 900 µg/day  Women   19-30 yr: 500 µg/day & 700 µg/day  31-50 yr: 500 µg/day & 700 µg/day  51-70 yr: 500 µg/day & 700 µg/day  >70 yr: 500 µg/day & 700 µg/day    **UL:**  **Infants**   0-12 months 600 µg/day  **Children and adolescents**   1-3 yr 600 µg/day  4-8 yr 900 µg/day  9-13 yr 1,700 µg/day  14-18 yr 2,800 µg/day  **Adults 19+ yr**   Men: 3,000 µg/day  Women : 3,000 µg/day  **Pregnancy**   14-18 yr: 2,800 µg/day  19-50 yr: 3,000 µg/day  **Lactation**   14-18 yr: 2,800 µg/day  19-50 yr: 3,000 µg/day | Nutrient Adequacy | Vitamin A |
| 11 | **Infants: (AI)**  0-6 months: 25 mg/day  7-12 months: 30 mg/day    **Children & adolescents: (EAR & RDI)**  1-3 yr: 25 mg/day & 35 mg/day  4-8 yr: 25 mg/day & 35 mg/day  Boys  9-13 yr: 28 mg/day & 40 mg/day  14-18 yr: 28 mg/day & 40 mg/day  Girls  9-13 yr: 28 mg/day & 40 mg/day  14-18 yr: 28 mg/day & 40 mg/day    **Adults: (EAR & RDI)**  Men  19-30 yr: 30 mg/day & 45 mg/day  31-50 yr: 30 mg/day & 45 mg/day  51-70 yr: 30 mg/day & 45 mg/day  >70 yr: 30 mg/day & 45 mg/day  Women  19-30 yr: 30 mg/day & 45 mg/day  31-50 yr: 30 mg/day & 45 mg/day  51-70 yr: 30 mg/day & 45 mg/day  >70 yr: 30 mg/day & 45 mg/day | Nutrient Adequacy | Vitamin C |
| 12 | **Infants: (AI)**  0-6 months: 65 µg/day (as folate)  7-12 months: 80 µg/day    **Children & Adolescents: (EAR & RDI)**  1-3 yr: 120 µg/day & 150 µg/day  4-8 yr: 160 µg/day & 200 µg/day  Boys   9-13 yr: 250 µg/day & 300 µg/day  14-18 yr: 330 µg/day & 400 µg/day  Girls   9-13 yr: 250 µg/day & 300 µg/day  14-18 yr: 330 µg/day & 400 µg/day    **Adults: (EAR & RDI)**  Men   19-30 yr: 320 µg/day & 400 µg/day  31-50 yr: 320 µg/day & 400 µg/day  51-70 yr: 320 µg/day & 400 µg/day  >70 yr: 320 µg/day & 400 µg/day  Women   19-30 yr: 320 µg/day & 400 µg/day  31-50 yr: 320 µg/day & 400 µg/day  51-70 yr: 320 µg/day & 400 µg/day  >70 yr: 320 µg/day & 400 µg/day | Nutrient Adequacy | Folate |
| 13 | **Infants: (AI)**  0-6 months: 210 mg/day  7-12 months:   270 mg/day   **Children & Adolescents: (EAR & RDI)**  1-3 yr: 360 mg/day & 500 mg/day  4-8 yr: 520 mg/day & 700 mg/day  Boys  9-11 yr: 800 mg/day & 1,000 mg/day  12-13 yr: 1,050 mg/day & 1,300 mg/day  14-18 yr: 1,050 mg/day & 1,300 mg/day  Girls  9-11 yr: 800 mg/day & 1,000 mg/day  12-13 yr: 1,050 mg/day & 1,300 mg/day  14-18 yr: 1,050 mg/day & 1,300 mg/day    **Adults: (EAR & RDI)**  Men  19-30 yr: 840 mg/day & 1,000 mg/day  31-50 yr: 840 mg/day & 1,000 mg/day  51-70 yr: 840 mg/day & 1,000 mg/day  >70 yr: 1,100 mg/day & 1,300 mg/day  Women  19-30 yr: 840 mg/day & 1,000 mg/day  31-50 yr: 840 mg/day & 1,000 mg/day  51-70 yr: 1,100 mg/day & 1,300 mg/day  >70 yr: 1,100 mg/day & 1,300 mg/day | Nutrient Adequacy | Calcium |
| 14 | **Infants: (AI)**  0-6 months: 0.2 mg/day  **(EAR & RDI)**  7-12 months: 7 mg/day & 11 mg/day    **Children & Adolescents: (EAR & RDI)**  1-3 yr: 4 mg/day & 9 mg/day  4-8 yr: 4 mg/day & 10 mg/day  Boys  9-13 yr: 6 mg/day & 8 mg/day  14-18 yr: 8 mg/day & 11 mg/day  Girls  9-13 yr: 6 mg/day & 8 mg/day  14-18 yr: 8 mg/day & 15 mg/day    **Adults: (EAR & RDI)**  Men  19-30 yr: 6 mg/day & 8 mg/day  31-50 yr: 6 mg/day & 8 mg/day  51-70 yr: 6 mg/day & 8 mg/day  >70 yr: 6 mg/day & 8 mg/day  Women  19-30 yr: 8 mg/day & 18 mg/day  31-50 yr: 8 mg/day & 18 mg/day  51-70 yr: 5 mg/day & 8 mg/day  >70 yr: 5 mg/day & 8 mg/day | Nutrient Adequacy | Iron |
| 15 | **Infants: (AI)**  0-6 months: 2.0 mg/day  **(EAR & RDI)**  7-12 months: 2.5 mg/day & 3 mg/day    **Children & Adolescents: (EAR & RDI)**  1-3 yr: 2.5 mg/day & 3 mg/day  4-8 yr: 3.0 mg/day & 4 mg/day  Boys  9-13 yr: 5 mg/day & 6 mg/day  14-18 yr: 11 mg/day & 13 mg/day  Girls  9-13 yr: 5 mg/day & 6 mg/day  14-18 yr: 6 mg/day & 7 mg/day    **Adults: (EAR & RDI)**  Men  19-30 yr: 12 mg/day; 14 mg/day  31-50 yr: 12 mg/day; 14 mg/day  51-70 yr: 12 mg/day; 14 mg/day  >70 yr: 12 mg/day; 14 mg/day  Women  19-30 yr: 6.5 mg/day; 8 mg/day  31-50 yr: 6.5 mg/day; 8 mg/day  51-70 yr: 6.5 mg/day; 8 mg/day  >70 yr: 6.5 mg/day; 8 mg/day | Nutrient Adequacy | Zinc |
| 16 | **Infants: (AI)**  0-6 months: 120 mg/day (5.2 mmol)  7-12 months: 170 mg/day (7.4 mmol)    **Children & Adolescents: (AI)**  1-3 yr: 200-400 mg/day (9-17 mmol)  4-8 yr: 300-600 mg/day (13-26 mmol)  9-13 yr: 400-800 mg/day (17-34 mmol)  14-18 yr; 460-920 mg/day (20-40 mmol)    **Adults: (AI)**  Men: 460-920 mg/day (20-40 mmol)  Women: 460-920 mg/day (20-40 mmol)    **Adults (SDT)**  Men 18+ yr: 2,000 mg/day (86 mmol)  Women 18+ yr: 2,000 mg/day (86 mmol)    **Infants (UL)**  1-3 yr: 1,000 mg/day (43 mmol)  4-8 yr: 1,400 mg/day (60 mmol)  **Children and adolescents (UL)**  9-13 yr: 2,000 mg/day (86 mmol)  14-18 yr: 2,300 mg/day (100 mmol) | Nutrient Adequacy | Sodium |
| 17 | All kind of vegetables, including legumes/beans are nutrient dense, low in kilojoules, and are a good source of minerals and vitamins (such as magnesium, vitamin C and folate), dietary fibre and a range of phytochemicals including carotenoids.    Especially colourful vegetables, there is a reduced risk of stroke and weight gain    Serve = 75g or 100-350kJ    Toddlers 1-2 yr: 2 to 3 serves  Boys/Girls 2-3 yr: 2.5 serves  Boys/Girls 4-8 yr: 4.5 serves  Boys 9-11 yr /Girls 9-18 yr: 5 serves  Boys: 12-18 yr: 5.5 serves    Men 19-50 yr: 6 serves  Men 51-70 yr: 5.5 serves  Men 70+ yr: 5 serves  Women 19+ yr: 5 serves  Women Lactating: 7.5 serves | Five Food Groups | Vegetables/ Legumes and beans |
| 18 | Whole fruits are a much better choice and are more filling.    Serve = 150g or 350kJ    Toddlers 1-2 yr: 0.5 serve  Boys/girls 2-3 yr: 1 serve  Boys/Girls 4-8 yr: 1.5 serves  Boys/Girls 9-18 yr: 2 serves    Men/Women 19+ yr: 2 serves | Five Food Groups | Fruit |
| 19 | Mostly wholegrain and/or high-fibre varieties.    Serve = 500kJ   Bread or roll (40g)  75-120g cooked rice, pasta, etc.  120g cooked porridge  30g cereal    Toddlers 1-2 yr: 4 serves  Boys 2-8 yr: 4 serves  Girls 2-13 yr: 4 serves  Boys 9-11 yr: 5 serves  Boys 12-13 yr: 6 serves  Girls 12-13 yr: 5 serves  Boys/Girls 14-18 yr: 7 serves  Girls pregnancy: 8 serves  Girls lactating: 9 serves    Men 19-70: 6 serves  Women 19-50 yr: 6 serves  Women 51-70 yr: 4 serves  Men 70+ yr: 4.5 serves  Women 70+ yr: 3 serves  Women pregnant: 8.5 serves  Women lactating: 9 serves | Five Food Groups | Grain (cereal) foods, mostly wholegrain and / or high cereal fibre varieties |
| 20 | Lean meat and poultry.    Serve = 500-600kJ    Toddlers 1-2 yr: 1 serve  Boys/Girls 2-3 yr: 1 serve  Boys/Girls 4-8 yr: 1.5 serves  Boys/girls 9+ yr: 2.5 serves  Girls Pregnant: 3.5 serves    Men 19-50 yr: 3 serves  Women 19-50 yr: 2.5 serves  Men 51+ yr: 2.5 serves  Women 51+ yr: 2 serves  Pregnant: 3.5 serves | Five Food Groups | Lean meat and poultry, fish, eggs, tofu, nuts and seeds and legumes/beans |
| 21 | Mostly reduced fat.    Serve = 500-600kJ  250ml milk  40g cheese  200g yoghurt    Toddlers 1-2 yr: 1-1.5 serves  Boys 2-3 yr: 1.5 serves  Girls 2-8 yr: 1.5 serves  Boys 4-8 yr: 2 serves  Boys 9-11 yr: 2.5 serves  Girls 9-11 yr: 3 serves  Boys/Girls 12-18 yr: 3.5 serves  Girls lactating: 4 serves    Men 19-70 yr: 2.5 serves  Women 19-50 yr: 2.5 serves  Women 51+ yr: 4 serves  Men 70+ yr: 3 serves | Five Food Groups | Milk, yoghurt, cheese and / or their alternatives (mostly reduced fat) |

**Supplement 3: Alignment with ESPEN hospital nutrition guidelines**^7^

| # | Recommendations | Mapping | |
| --- | --- | --- | --- |
|  |  | Main Category | Subcategory |
| 1 | Each hospital should have a list of available diets visible for patients and personnel. | Food Service Considerations | Menu Management System |
| 2 | Each hospital shall have a structured hospital food facility consisting of a kitchen, a delivery system, and an ordering system. | Food Service Considerations | Structure |
| 3 | Clear responsibilities for hospital food production and delivery are necessary for all areas of food supply | Food Service Considerations | Staffing |
| 4 | Hospitals should aim to use high-quality and sustainable food ingredients and to avoid food waste as much as possible. | Food Service Considerations | Sustainability |
| 5 | Patient and personnel surveys regarding hospital food and diets should be performed on a regular basis, at a minimum once a year. | Monitoring and Evaluation | Patient Satisfaction |
| 6 | Hospital food ordering should be structured, documented, and protocoled. | Food Service Considerations | Menu Management System |
| 7 | The prescription of hospital food should be performed through the computerised patient medical record. | Food Service Considerations | Menu Management System |
| 8 | Each hospital should propose a minimal number of two different regular diets (the standard and the hospital diets) and a minimal number of two different additional diets, adapted to the size and the focus of the hospital. | Patient Choice | Minimum Diets |
| 9 | Therapeutic diets should be only used if medically indicated. Otherwise, a regular diet should be used. | Special Diets | Therapeutic Diets |
| 10 | Diets based on food restriction without medical evidence (e.g. anticancer starvation) should be avoided in hospitals, because they increase the risk of malnutrition. | Special Diets | Therapeutic Diets |
| 11 | Hospital food diets should be re-evaluated every three to five years according to novel data in nutritional sciences and medicine, but also according to the hospital's focus and needs. | Monitoring and Evaluation | Menu review |
| 12 | Hospital nutrition should be checked, re-evaluated, and eventually adapted for each patient at regular intervals (every three to five days) according to the course of the disease, monitored oral intake, and the patient's acceptance. If dietary modifications are insufficient to cover energy and protein needs, medical nutrition should be provided according to the stage of the disease. | Monitoring and Evaluation | Clinical Nutrition Care |
| 13 | The hospitalised patients without or at low nutritional risk and who do not require special diets should be provided with the standard diet, as advised for the general population. The indication of this diet should be revaluated after three to five days. | Monitoring and Evaluation | Clinical Nutrition Care |
| 14 | Hospitalised patients at moderate/high nutritional risk or malnourished shall be provided with the hospital diet, a protein-energy enriched diet. | Patient Choice | Therapeutic Diets |
| 15 | The standard diet should cover the minimal energy needs (25 kcal/kg actual BW/day) and the minimum of protein needs (0.8-1.0 g/kg actual BW/day). The hospital diet should cover 30 kcal/kg actual BW/day of energy needs, and at least 1.2 g/kg actual BW/day of protein needs. | Nutrient Adequacy | Protein and Energy |
| 16 | The proportions of carbohydrates, lipids, and protein over the total daily energy intake should be 50-60%, 30-35%, and 15-20% for the standard diet, and 45-50%, 35-40%, and 20% for the hospital diet. | Nutrition Adequacy | Macronutrients |
| 17 | Hospitalised patients should be offered at least two menu choices for each main meal, lunch, and dinner. | Patient Choice | Minimum Choice |
| 22 | In patients with proven food allergies, the food allergen shall be excluded from the patient's hospital food choice and delivery. | Special Diets | Therapeutic Diets |
| 23 | Religious beliefs and food preferences (taste) should be taken into account at best when proposing the menu choice to the patient. | Patient Choice | Food Preferences |
| 24 | Vegetarian diets shall be designed to cover the energy and protein requirements | Patient Choice | Vegetarians |
| 25 | A vegan diet should not be offered at the hospital. | Patient Choice | Vegans |
| 26 | A gluten-free diet shall be provided to patients with proven celiac disease. | Special Diets | Therapeutic Diets |
| 27 | For individuals with irritable bowel syndrome, a diet low in fermentable oligo-, di-, monosaccharides, and polyols (low FODMAP diet) should be recommended to improve symptoms including abdominal pain and bloating and to increase the quality of life. | Special Diets | Therapeutic Diets |
| 28 | A diet low in lactose (<12 g per meal) shall be provided to patients with proven lactose intolerance (lactose breath test). | Special Diets | Therapeutic Diets |
| 29 | The hospital diet should be provided in the hospital setting to be served to malnourished patients, patients at risk for malnutrition, and other specific patient groups with a higher need for energy and/or protein. | Patient Choice | Minimum Diets |
| 30 | The specially designed hospital diet should be provided at the hospital because reaching the energy and/or protein target can hardly be realised with meals and snacking from the standard diet. | Special Diets | Therapeutic Diets |
| 31 | Hypocaloric diets are usually not indicated at the hospital and should be avoided because they increase the risk of malnutrition even in acute care obese patients. | Special Diets | Therapeutic Diets |
| 32 | There are very few indications for low-calorie diets in the hospital setting but they temporally can be indicated in refeeding syndrome, obesity with severe insulin resistance, and in rehabilitation units for obesity. | Special Diets | Therapeutic Diets |
| 33 | In low-calorie diets, the protein content may not be reduced and may be at least 1 g/kg actual BW/day if BMI is below 30, and at least 1 g/kg adjusted BW/day if BMI is greater than or equal to 30. | Special Diets | Therapeutic Diets |
| Liver disease | Protein intake should not be restricted in cirrhotic patients with hepatic encephalopathy as it increases protein catabolism. | Special Diets | Therapeutic Diets |
| Kidney disease | Chronic kidney disease patients previously maintained on controlled protein intake (the so-called “low protein diet”) should not be maintained on this regimen during hospitalization if acute illness is the reason for hospitalization | Special Diets | Therapeutic Diets |
| 34 | Patients with a proven chyle leakage should receive a diet low in long-chain triglycerides (LCT, <5% of total energy intake) and enriched in medium-chain triglycerides (MCT, >20% of total energy intake). | Special Diets | Therapeutic Diets |
| 35 | Patients with rare fatty acid oxidation disorders, such as long-chain 3-Hydroxyacil-CoA Dehydrogenase Deficiency (LCHADD, MIM 609016) and Mitochondrial Trifunctional Protein Deficiency  (MTPD, MIM 609015) and Very Long-Chain Acyl-CoA Dehydrogenase Deficiency (VLCADD, MIM 201475)* should receive a diet low in LCT (<5% of total energy intake) and enriched in MCT (>20% of total energy intake). | Special Diets | Therapeutic Diets |
| 36 | Some cases of intestinal lymphangiectasia with protein-losing enteropathy should receive a diet low in LCT (<5% of total energy intake) and enriched in medium-chain triglycerides (>20% of total energy intake). Energy and protein intakes should be at least 30 kcal/kg actual BW/day and 1.2 g/kg actual BW/day. | Special Diets | Therapeutic Diets |
| 37 | Neutropenic diets (also called “germ-free”, “no microbial” or “sterilised” diets) shall not be used (e.g. in neutropenic patients with cancer including hematopoietic cell transplant patients). | Special Diets | Therapeutic Diets |
| 38 | Solely on the day preceding a colonoscopy a low fibre diet should be eaten to achieve a better colon cleansing and to reduce patients’ discomfort. | Special Diets | Therapeutic Diets |
| 39 | In the case of chronic cardiac failure, chronic renal failure, or cirrhosis, sodium chloride reduction should not be decreased below 6 g/day, otherwise, the benefits-risk ratio is unfavourable towards a higher risk for malnutrition. | Special Diets | Therapeutic Diets |
| 40 | In case of arterial hypertension or acute decompensated heart failure, sodium chloride (salt) intake shall be no more than 6 g per day. | Special Diets | Therapeutic Diets |
| 41 | In patients admitted for acute decompensated heart failure, sodium should not be restricted to < 120 mmol/day (i.e. 2.8 g sodium chloride per day). | Special Diets | Therapeutic Diets |
| 42 | Patients treated with a short-term (6 weeks) systemic corticosteroid therapy may receive the hospital diet | Special Diets | Therapeutic Diets |
| 43 | Type 1 and 2 diabetic patients should be offered the standard or the hospital diet according to their nutritional risk/status | Special Diets | Therapeutic Diets |
| 44 | Patients with insulin therapy shall receive support to identify and quantify their dietary carbohydrate intake for glycaemic control. | Special Diets | Therapeutic Diets |
| 45 | Snacks containing mixed carbohydrates and protein should be offered between meals according to individual care (e.g. usually with mealtime short- and median-acting insulin) and glycaemic control. | Special Diets | Therapeutic Diets |
| 46 | In hospitalised diabetic patients, the low carbohydrate diet (<40% of energy intake) should be avoided as it is associated with lower energy intake and the risk of malnutrition. | Special Diets | Therapeutic Diets |
| 47 | With diabetic complications (e.g. diabetic nephropathy, diabetic gastroparesis, lower limb ulcers, and amputations), diet and nutrition support should be individual and diagnosis based. | Special Diets | Therapeutic Diets |
| Geriatrics | Older persons with malnutrition or at risk of malnutrition and signs of oropharyngeal dysphagia and/or chewing problems shall be offered texture- modified, enriched foods as a compensatory strategy to support adequate dietary intake. | Patient Choice | Texture Modified Diet |
| 48 | In clinical situations at risk of dysphagia (stroke, neurogenic and neuromuscular disorders, head and neck cancer, amyotrophic lateral sclerosis, hereditary ataxia, multiple sclerosis, or traumatic cervical spinal cord injury), systematic screening of dysphagia should be performed, and the need and type of modified texture diet should be identified. | Patient Choice | Texture Modified Diet |
| 49 | In the initial stages of dysphagia, adequate nutrition intake may be achieved through dietary modification to include soft, semisolid, or semi-liquid consistencies, in combination with appropriate swallowing techniques. | Patient Choice | Texture Modified Diet |
| Acute and chronic pancreatitis | Oral feeding shall be offered as soon as clinically tolerated and independent of serum lipase concentrations in patients with predicted mild acute pancreatitis. | Special Diets | Therapeutic Diets |
|  | Low-fat, soft oral diet shall be used when reinitiating oral feeding in patients with mild acute pancreatitis. | Special Diets | Therapeutic Diets |
|  | Patients with chronic pancreatitis do not need to follow a restrictive diet. | Special Diets | Therapeutic Diets |
|  | Chronic pancreatitis patients with a normal nutritional status should adhere to a well-balanced diet. | Special Diets | Therapeutic Diets |
|  | Malnourished patients with chronic pancreatitis should be advised to consume high protein, high-energy food in five to six small meals per day. | Special Diets | Therapeutic Diets |
|  | In patients with chronic pancreatitis, diets very high in fibre should be avoided. | Special Diets | Therapeutic Diets |
|  | In patients with chronic pancreatitis, there is no need for dietary fat restriction unless symptoms of steatorrhea cannot be controlled. | Special Diets | Therapeutic Diets |
| 50 | Small meals five to six times per day may help patients to tolerate oral feeding and achieve nutritional goals faster during the early phase of recovery after surgery | Special Diets | Therapeutic Diets |
| Surgery | In most instances, oral nutritional intake shall be continued after surgery without interruption. | Special Diets | Therapeutic Diets |
|  | It is recommended to adapt oral intake according to individual tolerance and to the type of surgery carried out with special caution to elderly patients. | Special Diets | Therapeutic Diets |
|  | Oral intake, including clear liquids, shall be initiated within hours after surgery in most patients. | Special Diets | Therapeutic Diets |
| 51 | After lower gastrointestinal bleeding, once oral food is authorised, patients should receive the standard hospital diet according to the patient nutritional risk and status. | Special Diets | Therapeutic Diets |
| 52 | After upper gastrointestinal system bleeding, once oral food is authorised, oral feeding should be initiated with liquids and advanced within 24 h to standard or hospital diet according to the patient nutritional risk and status. | Special Diets | Therapeutic Diets |
| 53 | Patients undergoing an endoscopic procedure should return to a standard hospital diet after the release of medication (anaesthesia) to prevent malnutrition risk during hospitalisation. | Special Diets | Therapeutic Diets |
| 54 | The combination of therapeutic diets may not be prescribed, as the risk of insufficient food intake and malnutrition is high. | Special Diets | Therapeutic Diets |
| 55 | Food intake is part of nutritional assessment and should be monitored by semi-quantitative methods at hospital admission, at least every week during the hospital stay in patients with no nutritional risk, and every day in patients with nutritional risk or malnourished. | Monitoring and Evaluation | Clinical Nutrition Care |
| 56 | In nutritionally at-risk patients, insufficient food intake equal to or less than 50% of energy requirements over 3 days during the hospital stay should trigger a nutritional intervention. | Monitoring and Evaluation | Clinical Nutrition Care |

**Supplement 4: Data extraction tool – categorisation matrix**


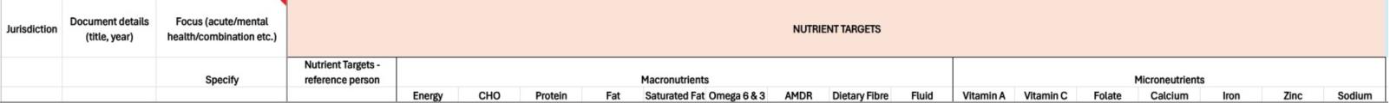


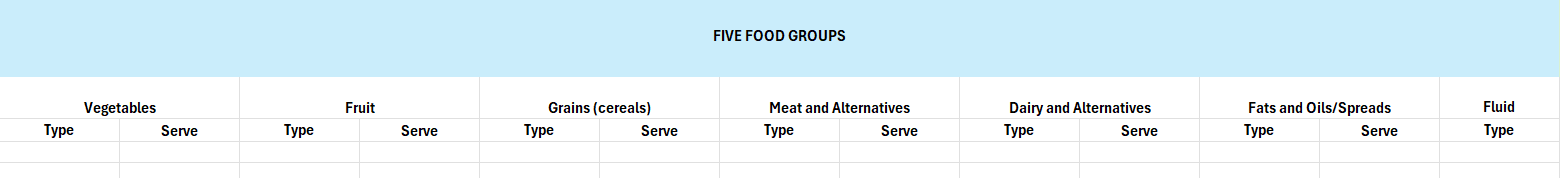


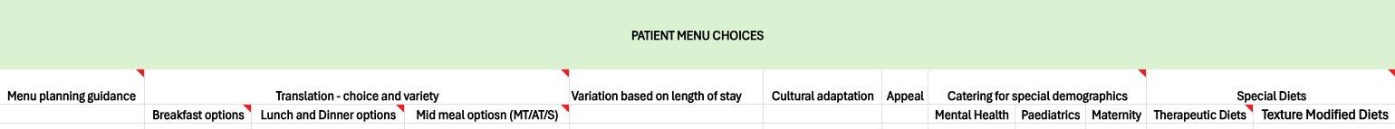


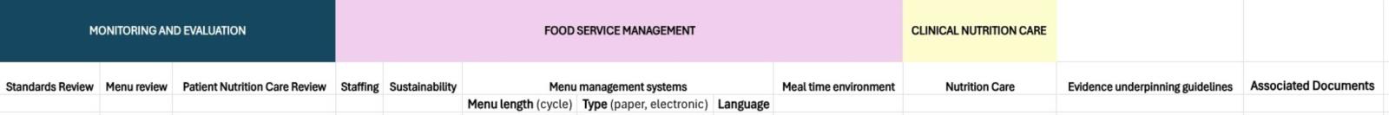


**Supplement 5: Coding framework**

| Code categories/domains | Source reference | Fully aligned | Partially aligned | Not aligned | Unable to determine |
| --- | --- | --- | --- | --- | --- |
| Five food groups | | | | | |
| Vegetables and legumes, Fruit, Grain (cereals) foods, Lean meats and poultry, fish, eggs, tofu, nuts and seeds, Dairy and alternatives | ADG | Includes **all** the 5 five food groups and **all of them** meet the minimum recommended serves for the hospital reference person. | Includes all/some of the 5 five food groups. **At least one but not all of them** meet the minimum recommended serves for the hospital reference person | Does not include **any** of the 5 five food groups **OR** include all/some but **none of them** meet the minimum recommended serves for the hospital reference person |  |
| Nutrient adequacy | | | | | |
| Macronutrient - Energy & Protein targets | ESPEN | Baseline/Standard diet meets **both** the minimal energy needs (25 kcal/kg actual BW/day) and the minimum of protein needs (0.8-1.0 g/kg actual BW/day). | Baseline/Standard diet meets **either** the minimal energy needs (25 kcal/kg actual BW/day) or the minimum of protein needs (0.8-1.0 g/kg actual BW/day). | No target mentioned OR only qualitative (e.g., “encourages protein”) |  |
| Macronutrient - proportion targets | ESPEN | Specifies **all** proportions of carbohydrates, lipids, and protein over the total daily energy intake and **all of them** are within the range of 50-60%, 30-35%, and 15-20% respectively for the standard diet (for those not at nutritional risk), and 45-50%, 35-40%, and 20% respectively for the hospital diet (for those at nutritional risk). | Specifies **all** proportions of carbohydrates, lipids, and protein over the total daily energy intake but only **some of them** are within the range OR only **some** (but not all) proportion of carbohydrates, lipids and proteins are specified and **at least one of them** is within the targeted range | Specifies some or all proportion of carbohydrates, lipids and proteins but none of them are within the targeted range | No lipids, carbohydrates, protein as proportion of total energy intake specified. |
| Macronutrient - Saturated Fats, Dietary Fibre, Fluids | ADG | Specifies **all** targets quantitatively for saturated fat, dietary fibre and fluid targets and **all of them** meets minimum recommendations for hospital reference person | Only specifies **some** targets quantitatively and/or **does not meet all** minimum recommendations for hospital reference person. | No targets mentioned OR only qualitative (e.g., “high fibre options”) |  |
| Micronutrient targets - Vitamin C, Folate, Calcium, Iron, Zinc, Sodium | ADG | Specifies **all** targets quantitatively (Vitamin C, Folate, Calcium, Iron, Zinc, Sodium) and meets minimum recommendations for hospital reference person. | Only specifies **some** targets quantitatively and/or **does not meet all** minimum recommendations for hospital reference person. | No targets mentioned OR only qualitative (e.g., “meets adequate needs”) |  |
| Patient Menu Choice | | | | | |
| Minimum choice main meals | ESPEN | Hospitalised patients offered **at least two** main menu choices for each main meal (Lunch and Dinner) | Any of the main meals has **less than two** minimum choice | No minimum choice for main meals specified. |  |
| mid meals | ESPEN | Systematic Mid Meal snacks offered daily as part of the meal service: **morning tea, afternoon tea** and **supper** daily (supper important to prevent night fasting). | **Less than three** mid meals offered daily | No systematic mid meal snack service |  |
| minimum diets | ESPEN | Offers a minimum two different regular diets - standard for those not at nutritional risk (min 25 kcal/kg actual BW/day and min protein 0.8-1.0 g/kg actual BW/day) and hospital diet (min 30 kcal/kg actual BW/day and min protein 1.2 g/kg actual BW/day) for those at nutritional risk PLUS a minimum two different additional diets (adapted to the size and the focus of the hospital). | Offers standard diet for those not at nutritional risk and the hospital diet for those at nutritional risk) BUT unable to determine/not specified hospital diet (for those at nutritional risk) meets min 30 kcal/kg actual BW/day min protein 1.2g/kg actual BW/day) AND/OR minimal number of other diets not specified or less than two. | Does not offer or offers less than two regular diets and additional diets |  |
| Therapeutic Diets Specifications (vegan and vegetarian) | ESPEN | Guidelines on development of therapeutic diets included/referenced in guidelines. Vegan diet are not offered at the hospital. Vegetarian diets mention energy and protein requirements to be met. | Mentions therapeutic diets but doesn't include or reference guidelines for these AND/OR Vegan diet offered AND/OR no requirements of energy or protein mentioned in vegetarian diets | Doesn't mention therapeutic diets AND Vegan diets offered **AND** energy and protein requirements not mentioned in vegetarian diets |  |
| Texture Modified Diets | ESPEN | TMD included in nutrition standards. Mentions screening for dysphagia but details of screening protocol beyond scope of this review. | TMD included in nutrition standards but screening protocols not mentioned/references in any way. | TMD not mentioned |  |
| Cultural Adaptations | ESPEN | Includes how to accommodate religious beliefs/food preferences (taste) in menu planning guidelines. | Mentions religious/food preferences in standards but doesn't consider it in menu planning guidelines specifically. | Doesn't mention/consider the religious/cultural aspect |  |
| Food Service Management | | | | | |
| Food Service Operations | ESPEN | Clear responsibilities for hospital food production and delivery stated **AND** states need for at least one dietitian dedicated to the hospital kitchen with the role of setting up patients’ menus according to the different available diets **AND** mentions aim to use high-quality and sustainable food ingredients and to avoid food waste as much as possible **AND** Hospital food delivery adapted to patient's abilities and perspectives (including acute care, rehabilitation unit, palliative care). AND mealtime should be protected | Mentions but unclear responsibilities for hospital food production and delivery stated AND/**OR** dietitian involved but their roles in the menu setting is unclear AND/**OR** does not mention either sustainable food ingredients **or** avoidance of food waste AND/**OR** mentions need for adaptation of food delivery to patient's abilities and perspectives, but does not specify acute care and/or rehab and/or palliative care. AND/OR mealtime should be protected | Staffing not mentioned **AND** no dietitian involved **AND** does not mention sustainability **AND** no mention of any need for mealtime delivery adaptations for different patient groups AND no mention of mealtime protection. |  |
| Menu Management System | ESPEN | Structured, documented and protocoled food ordering system mentioned. |  | Food ordering system not mentioned. |  |
| Monitoring and Evaluation | | | | | |
| Menu Assessment/Review | ESPEN | Patient and personnel surveys regarding hospital food and diets performed on a regular basis (minimum yearly). Hospital food diets should be re-evaluated every three to five years. | Menu is reviewed regularly but not within the specific timeframe. | Menu reviews not mentioned. |  |
| Patient Nutrition Care | ESPEN | Mentions and includes reference to document/guidelines on how patient's food intake is monitored during admission. Details on how food intake is monitored is beyond the scope of this review. | Mentions patient's food intake is monitored during admission but does not reference document/guidelines on protocols. | Does not mention or reference document/guidelines on monitoring of patient food intake during admission. |  |
| Clinical Nutrition Care Protocols | ESPEN | Specifies (or references document specifying) how patient nutrition should be checked, re-evaluated, and adapted at regular intervals, monitoring oral intake and the patient's acceptance. If dietary modifications are insufficient to cover energy and protein needs, medical nutrition intervention should be provided. | Mention of screening and/or nutrition assessment protocols but no specifics/no reference to document with more specific information. | No reference to screening/nutrition assessment protocols. |  |

**Supplement 6: Full electronic search strategy for all search engines included in review**

| **Search Engine** | **Date of search** | **Search** | **Limits applied** | **# results** | **# results pages screened** | **# results screened** | **# new records for full text review** | **# Duplicate** | **# not eligible*** |
| --- | --- | --- | --- | --- | --- | --- | --- | --- | --- |
| Google | 19-AUG-25 | (Hospitals OR inpatients OR institutions OR healthcare OR "health facility") AND (Nutrition OR menu OR food OR meals OR dining) AND (Standards OR Guidelines OR framework OR recommendations) | None for time. Region - Aus and language - English | 33 | 4 | 33 | 6 | 0 | 27 |
| Google Advanced | 19-AUG-25 | (Hospitals OR inpatients OR institutions OR healthcare OR "health facility") AND (Nutrition OR menu OR food OR meals OR dining) AND (Standards OR Guidelines OR framework OR recommendations) | Language: English  Region:  Australia  Last updated: Anytime  Site of Domain: .gov.au  Terms appearing anywhere in the page  File type: any format  Usage rights: not filtered by license | ~13,800,000 | 10 | 100 | 49 | 14 | 37 |
| health.tas.gov.au | 19-AUG-25 | nutrition standards | None | 19 | 2 | 19 | 0 | 1 | 18 |
| health.vic.gov.au | 20-AUG-25 | nutrition standards | None | 400 | 2 | 20 | 0 | 2 | 18 |
| sahealth.sa.gov.au | 20-AUG-25 | nutrition standards | None | 101 | 2 | 20 | 4 | 1 | 15 |
| health.nt.gov.au | 20-AUG-25 | nutrition standards | None | 864 | 2 | 20 | 0 | 0 | 20 |
| health.nsw.gov.au | 30-AUG-25 | nutrition standards | None | 2,830 | 2 | 20 | 3 | 3 | 14 |
| health.act.gov.au | 30-AUG-25 | nutrition standards | None | 1,323 | 2 | 20 | 0 | 0 | 20 |
| health.qld.gov.au | 30-AUG-25 | nutrition standards | None | 1,976 | 2 | 20 | 2 | 0 | 18 |
| health.wa.gov.au | 30-AUG-25 | nutrition standards | None | 50 | 2 | 20 | 0 | 0 | 20 |
| Trove | 26-AUG-25 | hospital nutrition standards | Language: English  Region: Australia  Category: Research and Reports | 1,116 | 10 | 100 | 4 | 5 | 91 |
| ACSQHC | 3-SEP-25 | hospital nutrition standards | None | 19 | 1 | 19 | 0 | 0 | 19 |

*Did not meet Concept (nutrition standards) and/or Context (hospital inpatients)

**Supplement 7: Sources excluded following full text review with reasons**

| Number | Search list | Document | Jurisdiction | Include (Y/N) | Rationale |
| --- | --- | --- | --- | --- | --- |
| 1 | Google | Patient Choice Standard for adult patients | NSW | N | Duplicate |
| 2 | GoogleAdv | Purpose and guiding principles \| Nutrition Standards | NSW | N | Duplicate |
| 3 | GoogleAdv | Promoting and supporting healthy food and drink choices | National | N | Out of scope: Context |
| 4 | GoogleAdv | Special considerations \| Nutrition Standards | NSW | N | Duplicate |
| 5 | GoogleAdv | Nutrition and quality food standards for adults in Victorian … | VIC | N | Duplicate |
| 6 | GoogleAdv | Introduction to Patient Choice Standard \| Nutrition Standards | NSW | N | Duplicate |
| 7 | GoogleAdv | Patient Meal Service, Food and Nutrition | NSW | N | Superseded |
| 8 | GoogleAdv | Healthy choices: policy directive and guidelines for health … | VIC | N | Out of scope: Context |
| 9 | GoogleAdv | Healthier food and drinks at healthcare facilities | QLD | N | Out of scope: Context |
| 10 | GoogleAdv | About the Nutrition Standards | NSW | N | Duplicate |
| 11 | GoogleAdv | Nutrition and quality food standards for adults in Victorian … | VIC | N | Duplicate |
| 12 | GoogleAdv | Nutrition and quality food standards for paediatric patients … | VIC | N | Duplicate |
| 13 | GoogleAdv | Healthy choices: policy guidelines for hospitals and health … | VIC | N | Out of scope: Context |
| 14 | GoogleAdv | 4. Nutrient banding and minimum menu choice tables | VIC | N | Duplicate |
| 15 | GoogleAdv | Appendix 8: Menu planning and Standards checklist | VIC | N | Duplicate |
| 16 | GoogleAdv | [Adult diet specifications](https://aci.health.nsw.gov.au/projects/diet-specifications/adult) | NSW | N | Duplicate |
| 17 | GoogleAdv | The bands \| Nutrition Standards | NSW | N | Duplicate |
| 18 | GoogleAdv | Nutrition Standards for Adult Patients | NSW | N | Duplicate |
| 19 | GoogleAdv | Nutrition Standards for Menu Items in Victorian Hospitals … | VIC | N | Superseded |
| 20 | GoogleAdv | Healthy Choices | VIC | N | Out of scope: Context |
| 21 | GoogleAdv | Nutrition Standards for Paediatric Patients | NSW | N | Duplicate |
| 22 | GoogleAdv | Food and Hotel Services - HealthShare NSW | NSW | N | Out of scope: Concept |
| 23 | GoogleAdv | Healthy eating strategies, policies and guidelines | VIC | N | Out of scope: Context |
| 24 | GoogleAdv | Diet supporting patient consumption - small meals - 6 day | NSW | N | Duplicate |
| 25 | GoogleAdv | review-food-standards-in-victorian-hospitals-and-aged- … | VIC | N | Out of scope: Concept |
| 26 | GoogleAdv | Sustainability \| Nutrition Standards | NSW | N | Duplicate |
| 27 | GoogleAdv | Background of adult diet specifications | NSW | N | Duplicate |
| 28 | GoogleAdv | Nutrition and quality food standards for paediatric patients … | VIC | N | Duplicate |
| 29 | GoogleAdv | Nutritious And Diverse Food For Victorian Hospitals | VIC | N | Out of scope: Concept |
| 30 | GoogleAdv | Guidelines for bringing occasional food to patients | NSW | N | Duplicate |
| 31 | GoogleAdv | Guidelines for Food Service to Vulnerable Persons | National | N | Out of scope: Concept |
| 32 | GoogleAdv | Healthy choices: policy directive for Victorian public health … | VIC | N | Out of scope: Concept |
| 33 | GoogleAdv | Nutrition Care - NSW Health | NSW | N | Duplicate |
| 34 | GoogleAdv | Review food standards in Victorian hospitals and aged care | VIC | N | Duplicate |
| 35 | GoogleAdv | Nutrition and quality food standards for adults in Victorian … | VIC | N | Duplicate |
| 36 | GoogleAdv | Nutrition standards : for adult inpatients in NSW hospitals … | NSW | N | Superseded |
| 37 | GoogleAdv | National and state policies | TAS | N | Out of scope: Context |
| 38 | GoogleAdv | Hospital-acquired complica | National | N | Out of scope: Concept |
| 39 | GoogleAdv | Nutrient Goal Standard for adult patients \| Nutrition Standards | NSW | N | Duplicate |
| 40 | GoogleAdv | Introduction to Nutrient Goal Standard \| Nutrition Standards | NSW | N | Duplicate |
| 41 | GoogleAdv | Sample minimum choices menu (paediatric) | NSW | N | Duplicate |
| 42 | GoogleAdv | health sector to lead in healthier food and drink choices for … | National | N | Duplicate |
| 43 | GoogleAdv | Nutrition and hydration | VIC | N | Out of scope: Context |
| 44 | GoogleAdv | Sample minimum choices menu (adult) \| Nutrition Standards | NSW | N | Duplicate |
| 45 | GoogleAdv | Guidelines for bringing occasional food to patients | NSW | N | Out of scope: Concept |
| 46 | SA health | Allied and scientific health standards, frameworks and guidelines | SA | N | Out of scope: Concept |
| 47 | SA health | Not your average meat and three veg - NALHNs approach to Parenteral Nutrition | SA | N | Out of scope: Concept |
| 48 | NSW health | Guidelines for the Inpatient Management of Adult Eating Disorders in General Medical and Psychiatric Settings in NSW | NSW | N | Out of scope: Concept |
| 49 | NSW health | Evidence, guidelines and reports | NSW | N | Duplicate |
| 50 | NSW health | NSW Eating Disorders Toolkit | NSW | N | Out of scope: Concept |
| 51 | QLD health | Queensland Health Nutrition Standards for Meals and Menus | QLD | N | Duplicate |
| 52 | QLD health | Queensland Health guidelines \| Queensland Health | QLD | N | Out of scope: Concept |

**Supplement 8: Broad overview of included documents**

| **Jurisdiction** | **Document Title** | **Year** | **Document Type** | **Target population** | **Previous version(s)** | **Endorsed by** |
| --- | --- | --- | --- | --- | --- | --- |
| Victoria | Nutrition and quality food standards for adults in Victorian public hospitals and residential aged care services | 2022 | Nutrition Standards | Adult inpatients | 2009 | Victoria Department of Health |
| Victoria | Nutrition and quality food standards for paediatric patients in Victorian hospitals | 2022 | Nutrition Standards | Paediatric inpatients | Nil | Victoria Department of Health |
| South Australia | Menu and Nutrition Standards for Public Health Facilities in South Australia | 2021 | Nutrition Standards | Inpatients | Created 2009, revised 2014 | South Australian Government |
| South Australia | Nutrition and Hydration Clinical Directive | 2019 | Policy Directive | Inpatients | Nil | South Australian Government |
| South Australia | Nutrition and Hydration Clinical Guideline | 2019 | Associated document | Inpatients | Nil | South Australian Government |
| Western Australia | Nutrition Standards for Adult Inpatients and Residential Aged Care Policy | 2020 | Nutrition Standards | Adult inpatients | Created 2012, reviewed 2018 | Western Australia County Health Service |
| Western Australia | Nutrition Screening, Assessment and Management Procedure | 2022 | Associated document | Adult and paediatric inpatients | Nil | Western Australia County Health Service |
| Queensland | Nutrition Standards for Meals and Menus | 2022 | Nutrition Standards | Adult and paediatric inpatients | 2012 | Nutrition and Menu Working Group, Statewide Foodservices |
| Queensland | Foodservice best practice  Queensland Health Guideline | 2023 | Associated document | Adult and paediatric inpatients | 2020 | Queensland Health |
| New South Wales | NSW Health Nutrition Standards | 2024 | Nutrition Standards | Adults and Paediatric inpatients | 2011 | Agency for Clinical Innovation |
| New South Wales | Diet Specifications | 2024 | Associated documents | Adults and paediatric inpatients | 2011 | Agency for Clinical Innovation |
| New South Wales | NSW Health Nutrition Care Policy Directive | 2017 | Policy Directive | Adults and paediatric inpatients | 2011 | Agency for Clinical Innovation |
| Australian Capital Territory | Nutrition Standards for Inpatients | 2019 | Nutrition Standards | Adults and paediatric inpatients | Nil | Australian Capital Territory Government |

Note: Links to Victoria Adult and Paediatric Nutrition Standards referenced on a single page, so they are recorded as 1 record retrieved (VIC n = 1). Australian Capital Territory refers to the New South Wales (NSW) nutrition standards so only the NSW standards were included in the comparative content analysis for subsequent phases of the review.

**Supplement 9: Comparison matrix rationale**

|  | **NSW (included ACI): Nutrition Standards, Diet Specifications, Nutrition Care** | | **QLD: Nutrition Standards and Food Service Best Practice Guidelines** | | **VIC: Adult and Paediatric Nutrition Standards** | | **SA: Nutrition Standards, Clinical Directive and Clinical Guidelines** | | **WA: Nutrition Standards, Nutrition Screening Management and Assessment Procedure** | |
| --- | --- | --- | --- | --- | --- | --- | --- | --- | --- | --- |
| **Domain** | **Align** | **Rationale** | **Align** | **Rationale** | **Align** | **Rationale** | **Align** | **Rationale** | **Align** | **Rationale** |
| **Five Food Groups** | **✓** |  | **✓** |  | **✓** | Adult and Paediatrics meet ADG CFG serves for reference age - if there is a difference in gender, higher serve is targeted regardless of ref gender (Adult male) | **✓** | Veg and fruit CFG min serves specified only. Mentions other food groups, and minimum choice specifications meet min recommended serves per day for ref person (6+ serves grains, 3+ serves meat and alt and 4+ serves dairy and alt options). Adult targets specified only.  Paeds refers to NSW ACI Nutrition Standards. | **✓** | Adult targets only, paeds out of scope. Veg and fruit CFG min serves specified only (Veg slightly lower ie 5 rather than 5.5 for reference male 19-50). Mentions other food groups, and minimum choice specifications meet min recommended serves per day for ref person (6+ serves grains, 3+ serves meat and alt and 4+ serves dairy and alt options) |
| **Nutrient Adequacy - macronutrients** | | | | | | | | | | |
| Energy + Protein | **✓** |  | **✓** | NOTE Long stay menu (patient admitted > 7 days OR at nutritional risk/malnourished) meet E + P targets for ESPEN baseline menu. Short Stay menu for patients/ not at nutritional risk/malnourished and admitted < 7 days below ESPEN standard diet Energy target of 105 kcal/kg/d | **✓** | Energy adult target above 25kcal/kg/d ref male 80kg. Paeds targets based on NRV upper age range male and PAL 1.2. Protein target above 0.8g/kg/d for adult and paeds. | **✓** | Adult targets specified only.  Paeds refers to NSW ACI Nutrition Standards. | **✓** | Adult targets only, paeds out of scope. |
| Proportion macronutrients as percent of total energy intake | **-** | Unable to determine as did not include target. | **-** | Unable to determine as did not include target. | **x** | No Paeds AMDR target specified. Adult AMDR aligns with ADG not ESPEN. | **x** | No targets specified for hospital population. Just notes total lipids may be different than general population target (20-35%) | **-** | No targets specified. Adult targets only, paeds out of scope. |
| Saturated fats + Dietary Fibre + Fluids | **✓** |  | P | Which did not align? Fluid requirements were not specified quantitatively | **✓** | Paeds also includes infant total fat g/d target and omega-3 and omega-6 targets (matches ADG) - infants only. | **✓** | Adult targets specified only.  Paeds refers to NSW ACI Nutrition Standards. | **✓** | Adult targets only, paeds out of scope. |
| **Nutrient Adequacy - micronutrients** | | | | | | | | | | |
| Vitamin C, Folate, Calcium, Iron, Zinc, Sodium | **✓** |  | **x** |  | **✓** | Also specifies Vitamin A RDI and UL for Paeds. | **✓** | Adult targets specified only.  Paeds refers to NSW ACI Nutrition Standards. Iron target in rationale notes 13mg (midpoint between reference Male 70+ of 8mg and reference female 19-50 of 18mg. Sodium target 2,300mg/d slightly above SDT - rationale is to ensure patients able to meet P/E targets (taste preferences). | **✓** | Iron target 11mg slightly higher than Male ref (8mg) and slightly lower than females of same age group (18mg). Technically does meet target for reference group (males 51-70 yrs). Adult targets only, paeds out of scope. |
| **Patient Choices** | | | | | | | | | | |
| Minimum choice main meals | **✓** |  | **✓** | NOTE Long stay menu (patient admitted > 7 days OR at nutritional risk/malnourished) meet minimum choice. Short Stay menu for patients. not at nutritional risk/malnourished and admitted < 7 days does not meet min 2 meal choice L/D/ | **✓** | ADULTS:  mains (L/D/): Min 2 choices/day (min one band 1, 1 hot) example menu shows 2 main choices at lunch and dinner. Also has min soup/sandwich options at L/D/ (with specified protein and energy targets. Paeds has min 2 main options/meal | **✓** | BF: min 1 hot cereal, 4 cold, 1 hot protein, 2 cold protein options; L/D: min 2 main choices/meal (1 must be group 1 or 2). Note this is adult only (Paeds: refers to ACI standards) | **✓** | Hot mains at least 2 choices at L/D/ |
| Mid meals | **✓** |  | **✓** | Notes in standards Patients/residents/clients can access at a minimum, three main meals and three mid-meals each day. Serve size variations are offered on the menu. | **✓** | Adults: 3 main meals/3 snacks/day  Paeds: infants 3 main meals and snacks on demand; Specifies no more than 14 hours between last meal service day/first meal service morning. Strongly recommends mid meals min 2/day. | **✓** | MT, AT, S. Note this is adult only (Paeds: refers to ACI standards) | **✓** | Snacks offered 3 times day |
| Minimum diets | **✓** | General full ward diet, HEHP (in TD), TMD (in TD), adaptations for Mental Health and short stay    Fully aligned as general full ward diet 105kj/kg/d and 1.2g protein/kg/d and High Protein diet is 120% full ward diet (1.4g/kg/d) - high energy does not specify kj but would be in addition to 105kj/kg/d so likely reach 125kj/kg/d hospital target. | P | Short stay (baseline diet 95-110kj/kg/d and protein 0.84g/kg/d) long stay (hospital diet 105-125kg/kg/d and protein 1.2-1.6g/kg/d) and TMD - no minimum TD diets but does include note TD broad category types and that should be selected based on  health population. Partial as long stay for those at nutritional risk did not have a minimum energy  target 125kj/kg/d and short stay for those not at nutritional risk did not have min energy target 105kj/kg/d | P | Partial as specifies 2 baseline diets (regular and texture modified 1005kj/kcal/d and 1.0 g/kg protein), with nutritionally at risk/malnourished patients extra requirements met by short order menu (and referral to dietitian advise how to select HEHP options and Short Order Menu but no protein or energy targets for this specified), but no specific HEHP diet noted on nutrition standards. Paediatric menu for children hospital. TD out of scope of these standards but does note that annual menu review needs to determine what TD health population of hospital required. | **✓** | Standard diet (105-125kj/kg/d and protein >1.2g/ kg/day) + HEP diet (would be above kj and protein targets of standard diet) + TMD + a la carte menu to supplement standard menu (short stay). TD no min specified (out of scope) but notes to refer to ACI for details. Note this is adult only (Paeds: refers to ACI standards) | **✓** | Standard diet (105-125kj/kg/d and protein >1.2g/ kg/day), HPHE diet for nutritionally at risk (and also extra options on standard diet so would be above protein/energy targets of standard diet), TMD (3 types + thickened fluid) also talks about allergies and Short Order Menu needs to be available. |
| Therapeutic Diets Specifications (vegan and vegetarian) | P | Partial as includes a vegan diet | P | Partial as TD mentioned but not reference to guidelines on how to determine/develop. States Vegetarian and Vegan options (with appropriate P/E requirements) to be included in short stay/long stay menus (not necessarily listed on patient menu, but available on master menu) so doesn't mention there should be an exclusive vegan menu, just that patients should be catered for. | P | Paeds: TD refers to ACI Diet Specifications but adult states out of scope, each hospital to determine which TD appropriate to provide based on population. No reference to any guidelines in adult included. Adult and Paed: Vegetarian option in menu and ensure plant-protein variety and note on vegan patients (refer to dietitian to ensure adequacy) does give tips on how to accommodate vegan/vegetarian options in main menu. | P | Ref ACI Therapeutic Diet specifications. Does include diets for vegans (and vegetarians - specifies needing to meet protein and energy requirements). Note this is adult only (Paeds: refers to ACI standards) | **✓** | Vegetarians catered for in standard menu when hospital identifies need to cater for vegetarian population (standard menu options need to meet min protein/energy as part of nutrition standards).  Vegan not mentioned. Details of Therapeutic Diets not accessible but are referenced (standards refer to local food service manuals/dietitian for these; associated document refers to TD details more explicitly. Assoc doc also does not refer specifically to catering to vegans. No where does it state there is not a vegan menu option as part of hospital policy |
| Texture Modified Diets | P | Partial as no screening info | P | Partial as no screening info | **✓** | TMD part of standard diet, mentions screening, SP etc just doesn't mention clinical areas need to focus on this in the standards. | **✓** | Doesn't mention areas of clinical risk but associated document (clinical guidelines) notes presence and severity of dysphagia identified as part of admission. Note this is adult only (Paeds: refers to ACI standards) | **✓** | States compliance to IDDSI and menu options specified. Assoc document references Speech assessment protocols. |
| Cultural/religious/Food preference adaptations | **✓** |  | **✓** | Includes in food service doc 3.2.1 how to do this in menu planning | **✓** | Both adult and paeds include cultural options as part of standard menu and in menu planning etc (major focus of menu design) | **✓** |  | P | No detail on this, just sentence referring to cultural, religious and social preferences should be considered in menu planning. |
| **Food Service Considerations** | | | | | | | | | | |
| Food Service Operations (Staffing, Sustainability, Mealtime) | P | Partial as roles/responsibilities is out of scope for the nutrition standards, and no details on Food Service Dietitian (nothing under 'staffing" NSW data extraction) | P | Partial as no detail on staff roles in either document. Does talk about dietitian role in menu planning etc. Does include information in sustainability and how to adapt for different patient groups - but doesn't mention palliative care. | P | Just doesn't mention palliative care (or where to go for this information) - and mental health is out of scope. Does advise how to and need to accommodate special patient groups (short stay/long stay/rehab) | P | Staffing roles mentioned generally (not very detailed) but food service dietitian role clearly specified. Specifies adaptation for different patient groups. Advanced meal care plans referenced in associated document (clinical guidelines). Sustainability not mentioned. | P | Sustainability not mentioned in standards or associated documents. Catering to different patient groups including long stay, mental health not covered. No reference on how to cater for these included. Says to refer to Food Service Manual/local dietitian for information in TDs. Palliative is mentioned in associated document (nutrition care planning) but rehab/long stay not specifically addressed. |
| Menu Management System | **✓** | In associated document | **✓** | Does go into detail on this in associated document. | - | Doesn't specify protocol. Mentions electronic meal ordering only (increases intake) | **✓** | Referenced in associated document (clinical guidelines) under food service management details. | - | Refers to Food Service Manual for information on diet ordering etc. Unable to access this - Unable to determine. |
| **Monitoring and Evaluation** | | | | | | | | | | |
| Menu Assessment/Review | P | Menu review every 2 years (compliance to standards) and mentions setting up system to get patient feedback on menu but no timeframe mentioned. | **✓** | Fully aligned as states full menu review by dietitian every 2 years and patient satisfaction with validated tool annually. | **✓** | Patient satisfaction quarterly and full menu reviews annually.  TD assessment/review part of menu planning and review checklist and  nutrition steering committee meets 6 times a year. | P | Full menu review once every 2 years. Notes need to make sure menu continuing to meet patient profile (i.e. diets) but doesn't specify timing for this or not clear if this forms part of this full menu review or separate. Separately mentions patient satisfaction questionnaire should be used, but no timing mentioned on this. States menu planning should include input staff and consumers. | **✓** | Specified annual compliance audit and customer satisfaction surveys. |
| Patient Nutrition Care | P | Monitoring intake mentioned in NCP associated documents but not details on how it is measured. | **x** |  | **x** | Details of screening/nutrition assessment guidelines not referenced in NS or associated documents. 'Nutrition and Hydration' online reference excluded in our full text search as not a standard/guideline or policy. | P | Monitoring patient food intake of those at nutritional risk mentioned in general terms | P | Food intake monitoring mentioned in associated documents but timing not specified. |
| Clinical Nutrition Care Protocol | **✓** |  | P | reference importance of screening etc nutritionally at risk patients, but doesn't reference document with more details/protocol on this | **x** | Details of screening/nutrition assessment guidelines not referenced in NS or associated documents. 'Nutrition and Hydration' online reference excluded in our full text search as not a standard/guideline or policy. | **✓** | Screening/nutrition assessment/NCP referenced in clinical policy/directive associated documents. | **✓** | NCP/screening protocols referenced in associated document. |

**✓**= fully aligned, **P** =partially aligned, **x** = not aligned, **-** = unable to be determine alignment. * Note: QLD short stay menu (for patients not at nutritional risk admitted for less than 7 days) energy targets and minimum choice and main meals were below ESPEN recommendations.

**Supplement 10: Preferred Reporting Items for Systematic reviews and Meta-Analyses extension for Scoping Reviews (PRISMA-ScR) Checklist**

| **SECTION** | **ITEM** | **PRISMA-ScR CHECKLIST ITEM** | **REPORTED ON PAGE #** |
| --- | --- | --- | --- |
| **TITLE** | | | |
| Title | 1 | Identify the report as a scoping review. | 1 |
| **ABSTRACT** | | | |
| Structured summary | 2 | Provide a structured summary that includes (as applicable): background, objectives, eligibility criteria, sources of evidence, charting methods, results, and conclusions that relate to the review questions and objectives. | ​​3 |
| **INTRODUCTION** | | | |
| Rationale | 3 | Describe the rationale for the review in the context of what is already known. Explain why the review questions/objectives lend themselves to a scoping review approach. | ​​5-6 |
| Objectives | 4 | Provide an explicit statement of the questions and objectives being addressed with reference to their key elements (e.g., population or participants, concepts, and context) or other relevant key elements used to conceptualize the review questions and/or objectives. | 6 |
| **METHODS** | | | |
| Protocol and registration | 5 | Indicate whether a review protocol exists; state if and where it can be accessed (e.g., a Web address); and if available, provide registration information, including the registration number. | ​​6 |
| Eligibility criteria | 6 | Specify characteristics of the sources of evidence used as eligibility criteria (e.g., years considered, language, and publication status), and provide a rationale. | ​​Supplement 1 |
| Information sources* | 7 | Describe all information sources in the search (e.g., databases with dates of coverage and contact with authors to identify additional sources), as well as the date the most recent search was executed. | ​6-7 |
| Search | 8 | Present the full electronic search strategy for at least 1 database, including any limits used, such that it could be repeated. | ​​Supplement 6 |
| Selection of sources of evidence† | 9 | State the process for selecting sources of evidence (i.e., screening and eligibility) included in the scoping review. | ​​7 |
| Data charting process‡ | 10 | Describe the methods of charting data from the included sources of evidence (e.g., calibrated forms or forms that have been tested by the team before their use, and whether data charting was done independently or in duplicate) and any processes for obtaining and confirming data from investigators. | ​8 |
| Data items | 11 | List and define all variables for which data were sought and any assumptions and simplifications made. | ​​8-9 |
| Critical appraisal of individual sources of evidence§ | 12 | If done, provide a rationale for conducting a critical appraisal of included sources of evidence; describe the methods used and how this information was used in any data synthesis (if appropriate). | ​​n/a |
| Synthesis of results | 13 | Describe the methods of handling and summarizing the data that were charted. | ​​9 |
| **RESULTS** | | | |
| Selection of sources of evidence | 14 | Give numbers of sources of evidence screened, assessed for eligibility, and included in the review, with reasons for exclusions at each stage, ideally using a flow diagram. | ​​9-10 |
| Characteristics of sources of evidence | 15 | For each source of evidence, present characteristics for which data were charted and provide the citations. | ​​Supplement 8 |
| Critical appraisal within sources of evidence | 16 | If done, present data on critical appraisal of included sources of evidence (see item 12). | ​​n/a |
| Results of individual sources of evidence | 17 | For each included source of evidence, present the relevant data that were charted that relate to the review questions and objectives. | ​​10-13 |
| Synthesis of results | 18 | Summarize and/or present the charting results as they relate to the review questions and objectives. | ​Table 1 and 2 |
| **DISCUSSION** | | | |
| Summary of evidence | 19 | Summarize the main results (including an overview of concepts, themes, and types of evidence available), link to the review questions and objectives, and consider the relevance to key groups. | 13-17 |
| Limitations | 20 | Discuss the limitations of the scoping review process. | 17-18 |
| Conclusions | 21 | Provide a general interpretation of the results with respect to the review questions and objectives, as well as potential implications and/or next steps. | 18 |
| **FUNDING** | | | |
| Funding | 22 | Describe sources of funding for the included sources of evidence, as well as sources of funding for the scoping review. Describe the role of the funders of the scoping review. | ​​n/a |

JBI = Joanna Briggs Institute; PRISMA-ScR = Preferred Reporting Items for Systematic reviews and Meta-Analyses extension for Scoping Reviews.

* Where *sources of evidence* (see second footnote) are compiled from, such as bibliographic databases, social media platforms, and Web sites.

† A more inclusive/heterogeneous term used to account for the different types of evidence or data sources (e.g., quantitative and/or qualitative research, expert opinion, and policy documents) that may be eligible in a scoping review as opposed to only studies. This is not to be confused with *information sources* (see first footnote).

‡ The frameworks by Arksey and O’Malley (6) and Levac and colleagues (7) and the JBI guidance (4, 5) refer to the process of data extraction in a scoping review as data charting*.*

§ The process of systematically examining research evidence to assess its validity, results, and relevance before using it to inform a decision. This term is used for items 12 and 16 instead of "risk of bias" (which is more applicable to systematic reviews of interventions) to include and acknowledge the various sources of evidence that may be used in a scoping review (e.g., quantitative and/or qualitative research, expert opinion, and policy document).

*From:* Tricco AC, Lillie E, Zarin W, O'Brien KK, Colquhoun H, Levac D, et al. PRISMA Extension for Scoping Reviews (PRISMAScR): Checklist and Explanation. Ann Intern Med. 2018;169:467–473. [doi: 10.7326/M18-0850](http://annals.org/aim/fullarticle/2700389/prisma-extension-scoping-reviews-prisma-scr-checklist-explanation).
